# Neoliensinine promotes lysosomal cell death in T cell malignancies via a novel STING-TUBA1B pathway

**DOI:** 10.1016/j.gendis.2025.101809

**Published:** 2025-08-14

**Authors:** Po Hu, Yuxuan Huang, Zipeng Yu, Xiaolong Zhang, Guangming Yang, Yang Pan

**Affiliations:** School of Pharmacy, Nanjing University of Chinese Medicine, Nanjing, Jiangsu 210023, China

**Keywords:** Cholesterol homeostasis, Lysosome-dependent cell death, Neoliensinine, Stimulator of interferon genes pathway, T cell malignancies

## Abstract

The clinical treatment options for T cell malignancies (T-CMs) remain limited, and even the current popular immunotherapies have yet to demonstrate significant efficacy in addressing this disease. Identifying effective therapeutic targets for T-CMs and developing corresponding drugs are still critical goals in this field. Stimulator of interferon genes (STING) is highly expressed in T cells and plays a pivotal role in T cell immunity. However, the mechanisms underlying STING-mediated cell death are not well understood. Neoliensinine (NeoL), a unique bisbenzylisoquinoline alkaloid from *Nelumbo nucifera* Gaertn, has been shown to be promising as a cytotoxic agent in this context. Here, we investigated NeoL and uncovered a novel mechanism linking STING-induced lysosome-dependent cell death in T-CMs. NeoL was found to bind to and activate STING, promoting its trafficking to lysosomes for degradation. This process was coordinated by tubulin alpha 1b (TUBA1B), a key microtubule isoform. The trafficking of STING to lysosomes disrupted cholesterol homeostasis, leading to lysosomal cholesterol accumulation. This, in turn, triggered lysosomal dysfunction, including lysosomal damage, membrane permeabilization, and cell death. Blocking any part of the STING-TUBA1B-lysosomal cholesterol accumulation axis attenuated NeoL-induced lysosomal disorders and T-CM cell death without significant effect on normal peripheral blood mononuclear cells. NeoL also decreased the growth of T-CM-derived tumors *in vivo*. These findings suggest that targeting STING-TUBA1B in T-CMs with NeoL is a promising therapeutic approach and provide a potential agent for improving T-CM therapy.

## Introduction

T cell malignancies (T-CMs) are characterized by the clonal evolution of cancerous T cells, resulting in conditions such as T cell leukemia or T cell lymphoma.^1^ At present, chemotherapy is the main treatment strategy for both adult and pediatric patients. However, the first-line treatment modalities achieve limited clinical responses in patients with T-CMs.^2^ Clinical drugs available for T-CM therapy are also limited, and nelarabine (a prodrug of Ara-GTP to inhibit DNA synthesis) was approved in 2005 for patients with relapsed/refractory acute T-lymphoblastic leukemia (T-ALL), and the complete response rate to nelarabine was only ∼30%.^3^^,^^4^

Despite the revolutionary impact of immunotherapy in cancer treatment, patients with T-CMs continue to respond poorly, with efficacy lower than that seen in B-cell malignancies.^2^^,^^5^ Research efforts are increasingly focused on optimizing immunotherapy for T-CMs, but challenges remain due to the lack of specific target antigens, the risk of nonspecific killing of normal T cells by chimeric antigen receptor-T cells, poor efficacy, and unavoidable side effects.^2^^,^^6^

The use of drug screening methods will lead to the search for personalized drugs for specific cancer types, and help discover potential drugs that cannot be detected under standardized treatment.^7^ Motivated by these insights, we believe that it is essential to continue exploring compounds sensitive to T-CMs and develop them for clinical application. When comparing alkaloids derived from *Plumula nelumbinis* (the embryo of *Nelumbo nucifera* Gaertn.) for T-CM treatment, we discovered that neoliensinine (NeoL), a tribenzylisoquinoline alkaloid, displayed a rapid and strong efficacy on inhibiting T-CMs.

Stimulator of interferon genes (STING), a master regulator in innate immune signaling, primes T cell activation and responses.^8^^,^^9^ Recent studies have uncovered additional functions of STING in cellular processes such as cell death,^10^ metabolism,^11^ non-classic autophagy, and inflammation.^12^ STING-mediated cell death, which can be triggered by compounds like cridanimod, vadimezan, or endogenous 2′,3′-cyclic GMP-AMP (cGAMP),^13^^,^^14^ has been observed. Although STING is reported to link to lysosomes or lysosome-dependent cell death,^10^ its precise regulatory mechanism remains unclear.

In this study, we discovered that NeoL facilitated STING degradation via the lysosome pathway and identified tubulin alpha 1b (TUBA1B) as a novel mediator of STING trafficking to lysosomes. We also showed that NeoL-induced STING trafficking to lysosomes promoted cholesterol accumulation on lysosomes, leading to lysosomal disorders and lysosome-dependent cell death of T-CMs. These findings revealed a novel link between NeoL and STING, established a non-classical function of STING in inducing cell death in T-CMs, and positioned NeoL as a potential therapeutic agent for T-CMs.

## Materials and methods

### Cell culture and reagents

Human T cell acute lymphoblastic leukemia cell lines (Jurkat and Molt4), T cell lymphoma cell lines (Hut102), diffuse large B cell lymphoma cell lines (Sudhl8, Sudhl4, R1.1, and OCILY3), mouse T cell lymphoma cell line (EL4), and human embryonic kidney cell line (293T) were obtained from the Cell Bank of Shanghai Institute of Biochemistry & Cell Biology (Shanghai, China). The identity of each cell line was confirmed by short tandem repeat (STR) profiling. All cell lines were regularly tested for *Mycoplasma* spp. infection using DAPI staining. Human peripheral blood mononuclear cells (PBMCs) from healthy donors were collected using lymphocyte-monocyte separation medium (Jingmei, Nanjing, China). PBMCs, Jurkat, Molt4, Hut102, Sudhl8, Sudhl4, R1.1, and OCILY3 cell lines were cultured in RPMI-1640 medium (GIBCO, Carlsbad, CA, USA), and EL4 and 293T cells were cultured in DMEM medium (GIBCO). Media were supplemented with 10% fetal bovine serum (FBS, GIBCO), 100 U/mL benzylpenicillin (Lukang, Jining, China), and 100 U/mL streptomycin (Lukang) in a humidified environment with 5% CO_2_ at 37 °C.

Neoliensinine (NeoL, MW 1028.26), liensinine (Lien, MW 610.74), isoliensinine (IsoL, MW 610.74), and neferine (Nef, MW 624.77), all were yellowish-white powders, and were provided by Dr. & Prof. Yang Pan of Nanjing University of Chinese Medicine. The purity of NeoL was at least 90%, while the other three alkaloids, Lien, IsoL, and Nef, had purities of at least 95%. The four compounds were isolated from *Plumula nelumbinis*, and their chemical structures were determined by ^1^H NMR, ^13^C NMR, and high-resolution mass spectrometry, and their purity was determined by HPLC peak area normalization.^15^ The molecular structure of NeoL is shown in our previous report.^15^ Bafilomycin A1 (BAF A1; KGATGR007) and LysoTracker RED (KGMP006) were purchased from KeyGen Biotech (Nanjing, China). Ammonium chloride (NH_4_Cl) was from HUSHI (Shanghai, China). 2′,3′-cGAMP (HY-100564), Dynasore (Dyn; HY-15304, an inhibitor of dynamin-dependent endocytosis), Ca-074 (HY-103350), colchicine (HY-16569), vinblastine sulfate (HY-13780), and H151 (HY-112693, STING inhibitor) were from MedChemExpress (Monmouth Junction, NJ, USA). BAPTA-AM (CSN10377) and atorvastatin (CSN12529) were from CSNpharm (Chicago, USA). Methylthiazolyldiphenyl-tetrazolium bromide (MTT, 40201ES72) was from YEASEN Biotech (Shanghai, China). Antifade mounting medium (P0126), antifade mounting medium with 4′,6-diamidino-2-phenylindole dihydrochloride (DAPI, P0131), 2′,7′-dichlorodihydrofluorescein diacetate (DCFH-DA; S0033S), and Fluo3-AM (S1056) were from Beyotime Biotechnology (Nanjing, China).

Antibodies for β-actin (AC026), heat shock protein 90 (HSP90, A5027), glyceraldehyde-3-phosphate dehydrogenase (GAPDH, A19056), light chain 3 (LC3, A17424; mouse mAb), TUBA1B (AC024), HSP70 (A23457), cathepsin B (CTSB; A19005), cathepsin D (CTSD; A19680), sequestosome 1 (P62/SQSTM1; A19700), TANK-binding kinase 1 (TBK1, A3458), and p-TBK1 (AP1418) were purchased from ABclonal Technology, Wuhan, China. p-STING (Ser365) (#72971), STING (#13647), lysosomal-associated membrane proteins 1 (LAMP1; mouse mAb #15665), and cleaved caspase-3 (#9664) were purchased from Cell Signaling Technology, Danvers, Massachusetts, USA. Antibodies for ubiquitin (10201-2-AP), cathepsin L (CTSL; 27952-1-AP), and LC3 (14600-1-AP) were from Proteintech Group, Chicago, Illinois, USA; and STING (ab208874) from Abcam. Secondary antibodies were horseradish peroxidase (HRP)-conjugated goat anti-rabbit IgG (H + L) (AS014), HRP-conjugated goat anti-mouse IgG (H + L) (AS003), HRP-conjugated mouse anti-rabbit IgG light chain (AS061), and HRP-conjugated goat anti-rabbit IgG heavy chain (AS064) from ABclonal Technology (Wuhan, China). Alexa Fluor 488 (A-11008 and A11001) and Alexa Fluor 594 (R37117 and A-11032) were from ThermoFisher Scientific, Waltham, Massachusetts, USA. For Western blotting, the dilution ratio of β-actin or GAPDH was 1:10,000, and the dilution ratio of HSP90 was 1:3000. The dilution ratio for other antibodies was 1:1000. As for immunofluorescence, the dilution ratio for antibodies was 1:400.

### Statistical analysis

All data were expressed as mean ± standard deviation from more than three independent experiments performed in parallel. One-way and two-way analysis of variance were used for statistical analysis of multiple column comparisons. The comparisons between two columns were analyzed using a two-tailed Student's *t*-test. *P* values were indicated on the graph, and *P* values less than 0.05 were considered statistically significant. The unpresented materials and methods can be found in the online supplementary files.

## Results

### Neoliensinine exerts favorable cytotoxicity against T cell malignancies

We first investigated the cytotoxicity of NeoL on three human T-CMs cell lines of Jurkat, Molt4, and Hut102, and compared it with abundant bisbenzylisoquinoline alkaloids in *Plumula nelumbinis*, including Lien, Nef, and IsoL. NeoL was shown to significantly suppress the viability of T-CM cell lines, with the average *IC*_50_ values of 9.89 ± 7.56 μmol/L at 6 h and 3.33 ± 1.41 μmol/L at 12 h, respectively. Other bisbenzylisoquinoline alkaloids in *Plumula nelumbinis* also exhibited cytotoxicity against T-CM cell lines, with the average *IC*_50_ values of 66.28 ± 17.24 μmol/L (Lien), 29.60 ± 9.06 μmol/L (Nef), and 23.31 ± 5.38 μmol/L (IsoL) for T-CMs at 24 h (Table 1). Thus, NeoL is the only abundant molecule that demonstrates superior efficiency over a shorter period against the survival of the T-CMs cells.

**Table 1:** The IC50 (μmol/L) of NeoL and abundant BBIs against cell lines of T cell malignancies.

Disease	Cell lines	NeoL				Lien		Nef		IsoL	
		6 h		12 h		24 h		24 h		24 h	
		*IC*50	95% CI	*IC*50	95% CI	*IC*50	95% CI	*IC*50	95% CI	*IC*50	95% CI
T-cell acute lymphoblastic leukemia	Jurkat	3.32	2.631 to 4.338	2.17	1.855 to 2.531	83.19	62.55 to 114.7	19.38	14.18 to 27.43	17.23	14.6 to 20.42
T-cell acute lymphoblastic leukemia	Molt4	18.15	11.07 to 40.21	4.89	4.182 to 5.749	66.91	58.59 to 76.57	32.76	28.14 to 38.17	25.23	19.75 to 32.45
T Cell lymphoma	Hut102	8.19	5.923 to 13.55	2.91	2.407 to 3.500	48.73	42.98 to 55.27	36.66	32.48 to 41.52	27.47	24.51 to 30.77
Mean ± S.D.		9.89±7.56		3.33±1.41		66.28±17.24		29.60±9.06		23.31±5.38	

NeoL: neoliensinine; Lien: liensinine; Nef: neferine; IsoL: isoliensinine

### Neoliensinine binds to STING and promotes its transient degradation

**Figure 1:**
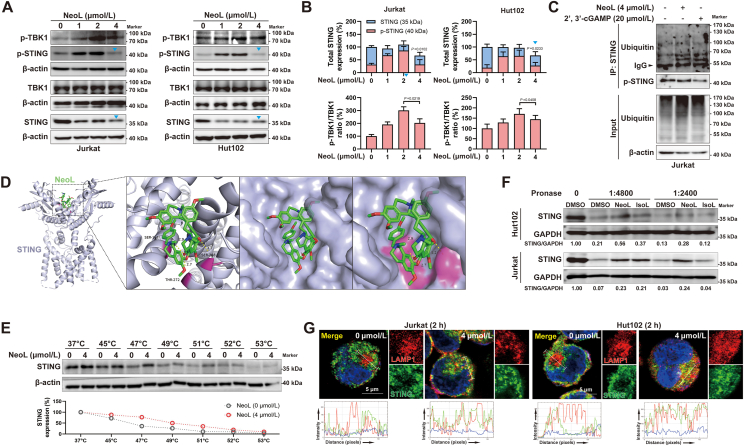
Neoliensinine binds to STING and promotes STING trafficking to lysosomes for degradation in T-cell malignancies. **(A)** NeoL activates STING-TBK1 pathways. Jurkat and Hut102 cells were treated with 0–4 μmol/L of NeoL for 4 h, and the expression of p-TBK1, TBK1, p-STING, and STING was determined by Western blotting, with β-actin as a loading control. p-STING and p-TBK1 were increased at 1 and 2 μmol/L but declined at 4 μmol/L (blue arrowheads). **(B)** Quantification of total expression of STING in Jurkat and Hut102 cells, showing significant reduction of total STING and p-STING (blue arrowheads) after 4 h of NeoL treatment at 4 μmol/L, suggesting accelerated degradation of p-STING under 4 μmol/L of NeoL (*n* = 3). **(C)** STING-Ubiquitin co-precipitation. Jurkat cells were treated with 20 μmol/L of 2′,3′-cGAMP for 12 h or 4 μmol/L of NeoL for 2 h. STING served as a loading control for immunoprecipitation. For input, β-actin served as a loading control, showing increased ubiquitin after STING activation. **(D)** The interaction between STING and NeoL was revealed by molecular docking. **(E)** The interaction between STING and NeoL was confirmed by DARTS assay. Cell lysates were incubated with DMSO, NeoL, or IsoL, and digested with pronase in 1:4800 or 1:2400 dilution. Immunoblotting demonstrated increased STING after NeoL incubation. GAPDH was used as a loading control. **(F)** The interaction between STING and NeoL was also confirmed by CETSA assay. Cells were incubated with 4 μmol/L of NeoL or DMSO for 30 min, divided into seven pairs, and treated at the indicated temperatures for 3 min. Soluble proteins were extracted by freezing/thawing in liquid nitrogen, blotted for STING or β-actin as a loading control. Increased STING was detected in lysate at 47 °C, 49 °C, and 51 °C. **(G)** The Jurkat and Hut102 cells were treated without or with 4 μmol/L of NeoL for 2 h and immune-labelled with anti-STING (green) and anti-LAMP1 for lysosomes (red). NeoL treatment increased the colocalization of lysosomes and STING. The coincidence of fluorescence curves was analyzed by ImageJ software. *P* values were indicated in the graph, and *P* < 0.05 indicates statistical significance.

T-CMs are a type of malignant tumor originating from T lymphocytes. The STING pathway is essential in T lymphocytes and acts as a major player in anti-tumor immunity.^16^ We first determined the STING expression level by immunoblotting and showed that STING was strongly expressed in three T-CM cell lines (Jurkat, Hut102, and Molt4), but was merely detectable in the four B-cell malignancy cell lines (Sudhl8, Sudhl4, R1.1, and OCILY3) (Fig. S1). This is consistent with the STING expression pattern in the normal T (70.2 nTPM) and B cells (5.1 nTPM) in the public database (https://www.proteinatlas.org/ENSG00000184584-STING1/single+cell). STING pathway was then determined in Jurkat (T cell leukemia) and Hut102 (T cell lymphoma) cells with NeoL. Phosphorylation of TBK1, the downstream target of the STING pathway, was increased under 1 or 2 μmol/L of NeoL treatment for 4 h. Meanwhile, p-STING (Ser365) was also enhanced (Fig. 1A). This suggested that NeoL could activate the STING pathway. It should be noted that STING, after phosphorylation, separates from the prototype STING on the Western blotting band.^17^ Therefore, the total STING expression shall combine the 35 kDa STING and 41 kDa p-STING. Although 1 or 2 μmol/L NeoL decreased the expression of STING (35 kDa), the protein quantification showed that the total STING expression was stable (Fig. 1B). However, after 4 μmol/L NeoL treatment for 4 h, the total expression of STING was decreased significantly (blue arrowhead; Fig. 1A and B), and 4 μmol/L of NeoL was therefore used for most experiments.

STING has been reported to undergo degradation after activation^18^; thus, we treated Jurkat cells with 4 μmol/L of NeoL for 2 h or with 20 μmol/L of a natural agonist of STING, 2′,3′-cGAMP, for 12 h, and compared the interaction of ubiquitin and STING by co-immunoprecipitation. Increased anti-ubiquitin reactivity was detected in anti-STING precipitates from both 2′,3′-cGAMP (12 h) and NeoL (2 h)-treated cells (Fig. 1C), indicating NeoL or 2′,3′-cGAMP primed STING to ubiquitin-mediated degradation. Under 20 μmol/L of 2′,3′-cGAMP, p-TBK1 and p-STING were increased in a time-dependent manner over 24 h, while the total STING was unchanged (Fig. S2). This suggests that NeoL could activate STING and induce efficient and transient STING degradation at lower concentrations and in shorter periods compared with 2′,3′-cGAMP.

We further investigated whether this was caused by direct binding of NeoL and STING via three strategies, molecular docking, cellular thermal shift assay (CETSA), and drug affinity responsive target stability (DARTS). Using molecular docking, we showed that the binding free energy of NeoL with STING was −8.4 kcal/mol, with Ser-167, Thr-272, and Ser-268 identified as possible binding sites (Fig. 1D).

CETSA is a label-free method that is extensively used to measure compound-target engagement in intact cells and tissue. Ligand-bound proteins display increased thermal stability and remain in solution under high temperature, which can be quantified by Western blotting, whereas unbound proteins denature and precipitate at elevated temperatures.^19^ We incubated 2 × 10^6^ cells with 4 μmol/L of NeoL or dimethyl sulfoxide (DMSO) for 30 min, divided them into seven parts, and treated them for 3 min at 37 °C, 45 °C, 47 °C, 49 °C, 51 °C, 52 °C, and 53 °C, respectively. Soluble protein extracts were immunoblotted with anti-STING antibody, and increased contents of STING were detected in the presence of NeoL at 47 °C, 49 °C, and 51 °C, supporting that NeoL binds to STING (Fig. 1E).

DARTS assay is based on the principle that binding of a ligand can protect its target protein from protease degradation. We equally divided the lysate from 1 × 10^7^ cells into seven groups, and incubated them with DMSO or 40 μmol/L of NeoL or 80 μmol/L of IsoL at 4 °C overnight, and digested at 37 °C for 30 min with pronase at 1:4800 or 1:2400 dilution. Immuno-blotting showed that incubation with NeoL and IsoL led to increased STING in the Jurkat and Hut102 cell lysates, and STING was more resistant to pronase degradation after NeoL treatment (Fig. 1F). These results together convincingly demonstrate that NeoL binds to STING directly. Because NeoL displayed higher binding affinity to STING and stronger inhibitory effects on T-CMs than IsoL, we hypothesized that NeoL induced T-CM cell death by promoting STING degradation.

### Neoliensinine promotes TUBA1B-coordinated STING trafficking to lysosomes for degradation in T cell malignancies

It has been reported that STING can be degraded via the lysosome pathway through ESCRT-driven microautophagy into LAMP1-positive compartments.^18^ To determine whether NeoL promoted STING for lysosomal degradation in T-CMs, we carried out double immuno-staining for STING and LAMP1 (a lysosomal membrane protein) in Jurkat and Hut102 cells (Fig. 1G). In the absence of NeoL, co-localization of STING (green; Fig. 1G) and lysosomes (red; Fig. 1G) was rarely detected. After treating T-CM cells for 2 h with 4 μmol/L of NeoL, significant colocalization of STING and LAMP1 was observed in both cell lines, indicating that NeoL indeed promotes STING trafficking to lysosomes.

**Figure 2:**
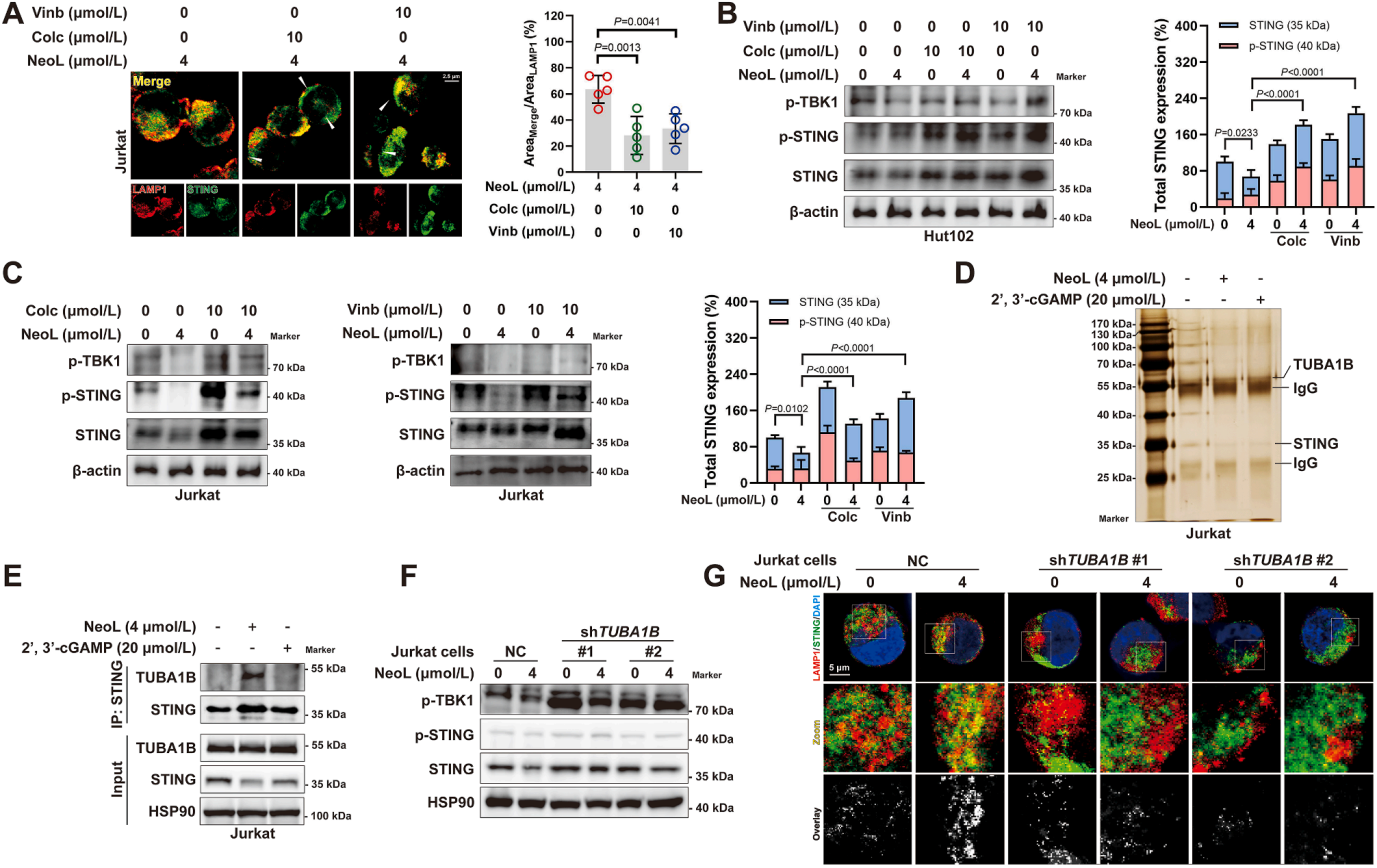
Microtubules and TUBA1B coordinate neoliensinine-induced STING trafficking to lysosomes for degradation in T-cell malignancies. **(A)** Jurkat cells were pre-treated for 1 h with microtubule destabilizers, 10 μmol/L of colchicine (Colc) or vinblastine sulfate (Vinb), and then with 4 μmol/L of NeoL for 4 h. Cells were immuno-labeled for LAMP1 (red) and STING (green) and imaged by confocal laser scanning microscopy. The levels of co-localization were quantified (*n* = 5). Microtubule destabilizers were shown to disrupt STING/LAMP1 co-localization. **(B, C)** Hut102 (B) and Jurkat (C) cells were pre-treated for 1 h with 10 μmol/L of Colc or Vinb, and then with 4 μmol/L of NeoL for 4 h. Protein extracts were immunoblotted for p-TBK1, p-STING, STING, or β-actin as a loading control (*n* = 3). The expression of p-STING and STING was quantified by Image J. Microtubule destabilizers were shown to increase the contents of total and p-STING. **(D)** Jurkat cells were treated without or with 20 μmol/L of 2′,3′-cGAMP for 12 h or 4 μmol/L of NeoL for 2 h. The cell lysates were immunoprecipitated with anti-STING, and the precipitates were resolved on SDS-PAGE for silver staining. A band at approximately 55 kDa was increased following 2′,3′-cGAMP or NeoL treatment. Proteomic assay of the precipitates showed that TUBA1B was enriched after NeoL treatment. **(E)** Jurkat cells were treated without or with 20 μmol/L of 2′,3′-cGAMP for 12 h or 4 μmol/L of NeoL for 2 h. The lysates were immunoprecipitated with anti-STING. The precipitates were resolved on SDS-PAGE and blotted with anti-TUBA1B. β-actin was used as a loading control for precipitates. For input, cell lysates were immunoblotted for TUBA1B, STING, or HSP90 as a loading control. TUBA1B was specifically detected in the NeoL-treated anti-STING precipitates. **(F)***TUBA1B* knockdown led to hyperactivation of the STING-TBK1 pathway. Jurkat cells were transfected with *TUBA1B* shRNA #1 or #2, and then treated without or with 4 μmol/L of NeoL for 4 h. β-actin was used as a loading control. Cell lysates were immunoblotted for p-TBK1, p-STING, STING, or HSP90 as a loading control. Significantly increased p-TBK1 contents showed hyperactivation of the STING-TBK1 pathway in *TUBA1B* knockdown cells. **(G)***TUBA1B* knockdown attenuates STING/LAMP1 co-localization. Jurkat cells were transfected with *TUBA1B* shRNA #1 or #2, and treated with 4 μmol/L of NeoL for 2 h. They were then processed for double immunostaining of LAMP1 and STING. Overlay indicates the colocalization signal of LAMP1 and STING (yellow). In normal cells (NC), STING was well co-localized with LAMP1 after NeoL treatment, and this was largely abolished in *TUBA1B* knockdown cells. *P* values were indicated in the graph, and *P* < 0.05 indicates statistical significance.

Microtubules are the components of the cytoskeleton, and they form an orbital system within the cell, facilitating the transportation of various molecules and organelles.^20^ Microtubule transport is closely related to autophagy degradation.^21^ Therefore, we pre-treated T-CM cells for 1 h with microtubule destabilizers (colchicine and vinblastine sulfate) and then treated cells for 4 h with 4 μmol/L of NeoL to examine the effects of microtubules on NeoL-induced STING trafficking. By assessing the co-localization of STING (green) and lysosomes (red), we found that colchicine or vinblastine suppressed NeoL-primed STING trafficking to lysosomes (Fig. 2A). STING contents were then quantified by immunoblotting, and microtubule destabilizers significantly increased both total STING and p-STING in Hut102 and Jurkat cells, suggesting that microtubules are crucial for NeoL-induced STING degradation (Fig. 2B and C). Interestingly, treatment with colchicine did not increase STING transcription, whereas vinblastine reduced STING mRNA expression (Fig. S3). This suggests that although STING degradation is an ongoing process in T-CMs, NeoL could promote STING degradation via the microtubule-lysosome pathway.

To further identify the regulator of NeoL-induced lysosomal trafficking of STING, we conducted co-immunoprecipitation-liquid chromatography/mass spectrometry to characterize potential factors interacting with STING. Jurkat cells were treated with NeoL for 2 h or 2′,3′-cGAMP for 12 h, the cell lysates were precipitated with anti-STING, and the precipitates were resolved on SDS-PAGE. Silver staining of the SDS-PAGE showed an increased protein band at approximately 55 kDa (Fig. 2D). Proteomic analysis revealed that TUBA1B was the only STING-interacting microtubule molecule among the top 20 proteins in the STING precipitates after NeoL treatment. For 2′,3′-cGAMP, it was TUBA1A that co-precipitated with STING (Table S1–S3).

TUBA1A/B are important microtubule isoforms that are involved in the formation of the cytoskeleton.^22^ We then performed co-immunoprecipitation, and TUBA1B was strongly detected in anti-STING precipitates specifically from NeoL-treated but not 2′,3′-cGAMP-treated cells (Fig. 2E). To demonstrate the role of TUBA1B in coordinating NeoL-induced STING degradation, three shRNAs were used to knock down *TUBA1B* (Fig. S4). In sh*TUBA1B*-treated cells, STING degradation was inhibited, and consequently, increased p-TBK1 was observed (Fig. 2F), which correlated with enhanced STING activation (Fig. S5). The colocalization of STING and lysosomes was also disrupted in sh*TUBA1B* cells (Fig. 2G). These results showed that in NeoL-treated T-CMs, TUBA1B coordinated STING degradation via the microtubule-lysosome pathway.

### Neoliensinine induced-STING trafficking to lysosomes mediated lysosomal cholesterol accumulation

A previous study revealed that STING is recruited by NPC intracellular cholesterol transporter 1 (NPC1) for lysosomal degradation.^23^ NPC1 is a membrane protein located on the late endosome or lysosome that removes cholesterol.^24^ Loss of the cholesterol exporter, NPC1, causes cholesterol accumulation within lysosomes.^25^ As NeoL induced transient and abundant STING degradation, we speculated that STING degradation might interfere with the removal of cholesterol from lysosomes.

**Figure 3:**
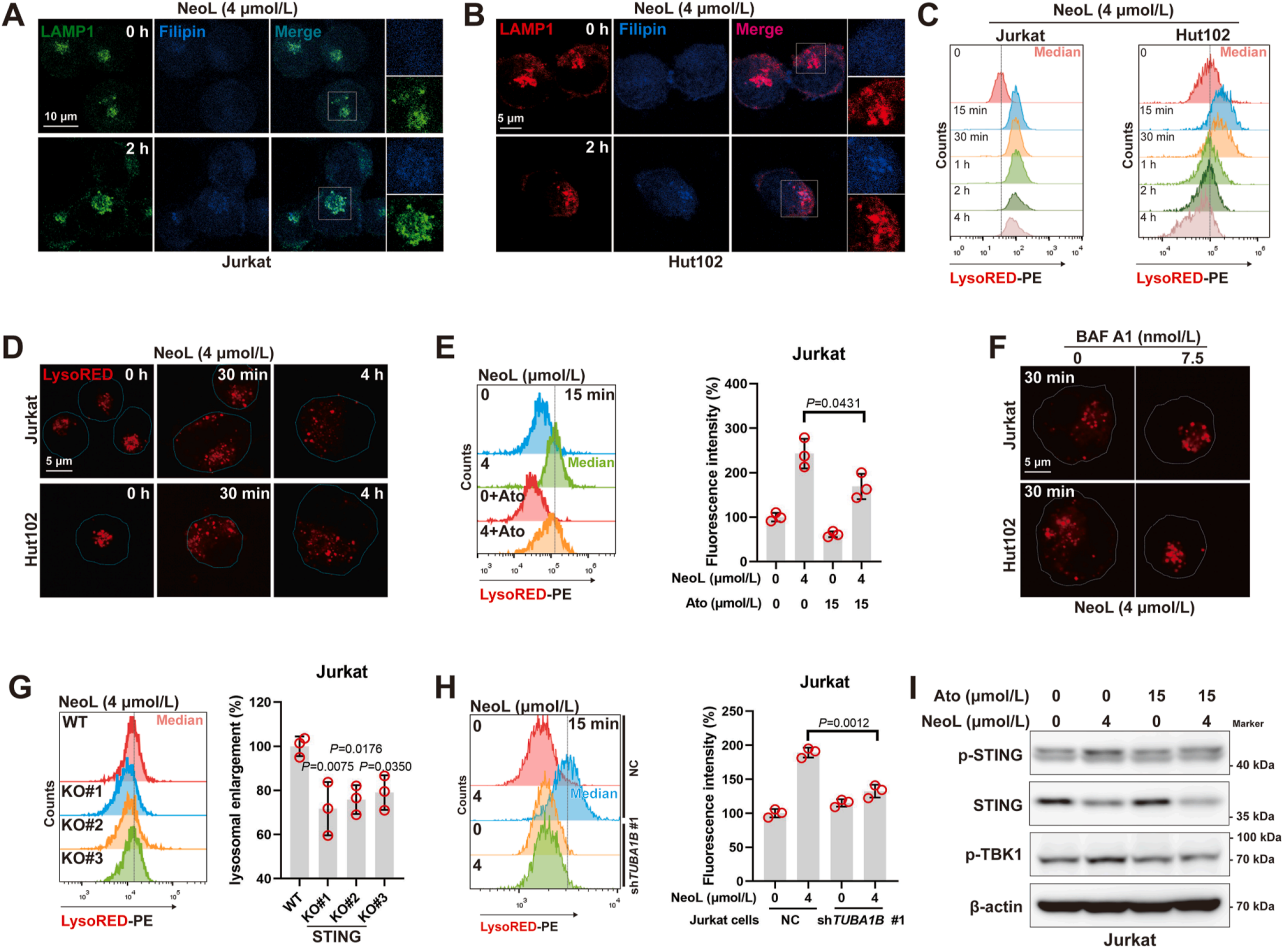
Neoliensinine induces STING-dependent cholesterol accumulation on lysosomes in T-cell malignancies. **(A, B)** Jurkat or Hut102 cells were treated with 0 or 4 μmol/L of NeoL for 2 h, processed with LAMP1 for lysosomes (green in A or red in B) and Filipin III staining for cholesterol (in blue), and imaged by confocal laser scanning microscopy. The increased colocalization was detected after NeoL treatment, showing cholesterol accumulation in lysosomes after NeoL treatment. **(C)** Jurkat or Hut102 cells were treated without or with 4 μmol/L of NeoL for 15 min, 30 min, 1 h, 2 h, or 4 h, and lysosomes were stained with LysoTracker RED. The fluorescence intensity was detected by flow cytometry. Lysosomal enlargement was observed, as the LysoTracker RED signals shifted to the right after NeoL treatment. **(D)** Jurkat or Hut102 cells were treated without or with 4 μmol/L of NeoL for 30 min or 4 h. Lysosomes were stained with LysoTracker RED and imaged by confocal microscopy, showing lysosomal enlargement after NeoL treatment. The dotted green outlines were artificially marked for the cell boundary, which was determined by increasing the contrast of the image. **(E)** Jurkat cells were pre-treated with 15 μmol/L of atorvastatin (Ato), a blocker of cholesterol production, for 6 h and then treated with 4 μmol/L of NeoL for 15 min. Cells were stained with LysoTracker RED and subjected to flow cytometry (*n* = 3). Ato was shown to partially reduce lysosomal enlargement. **(F)** Jurkat or Hut102 cells were pre-treated without or with 7.5 nmol/L bafilomycin A1 (BAF A1) for 1 h to block trafficking and then treated with 4 μmol/L of NeoL for 30 min. Cells were stained with LysoTracker RED and imaged by confocal microscopy. The dotted white outlines artificially mark cell edges by increasing the image contrast. BAF A1 was shown to largely reverse NeoL-induced lysosome distribution. **(G)** STING KO Jurkat cells (#1, #2, and #3) were treated without or with 4 μmol/L of NeoL for 15 min, stained with LysoTracker RED, and quantified by flow cytometry (*n* = 3). STING KO was shown to attenuate lysosomal enlargement **(H)** Jurkat cells were transfected with *TUBA1B* shRNA #1 and treated with 4 μmol/L of NeoL for 15 min and stained with LysoTracker RED (*n* = 3). The lysosomal enlargement was seen in normal cells (NC) but was largely abolished by *TUBA1B* knockdown. **(I)** Jurkat cells were pre-treated with 15 μmol/L of Ato for 6 h and then treated with 4 μmol/L of NeoL for 4 h. Expression of p-STING, STING, and p-TBK1 was determined by Western blotting with β-actin as a loading control. Ato was shown to cause NeoL-induced TBK1 activation. *P* values were indicated in the graph, and *P* < 0.05 indicates statistical significance.

Firstly, we tested whether NeoL treatment induced cholesterol accumulation on lysosomes, by labelling lysosomes with anti-LAMP1 (green or red) and cholesterol with Filipin staining (blue).^26^ NeoL was shown to promote cholesterol deposition in lysosomes of T-CMs cells (Fig. 3A and B). Meanwhile, LysoRED fluorescent probe was used to label lysosomes in live cells. Lysosomal cholesterol accumulation can enlarge lysosomes, and increased LysoRED fluorescent intensity can be detected.^27^ Here, we detected enlargement of lysosomes within 15 min of NeoL induction (Fig. 3C). Cholesterol accumulation also appeared to influence the distribution of lysosomes within the cell, and aggregated lysosomes became dispersed under the treatment of NeoL (Fig. 3D).

To determine whether these subcellular alterations were directly caused by cholesterol accumulation, we pre-treated Jurkat cells for 6 h with atorvastatin, an inhibitor of HMG-CoA reductase to block cholesterol production, and then treated the cells with NeoL for 15 min. NeoL-induced lysosomal enlargement was largely suppressed by atorvastatin pre-treatment (Fig. 3E; Fig. S6). These data suggested that NeoL promoted cholesterol accumulation in lysosomes.

We subsequently evaluated whether NeoL-induced lysosomal cholesterol accumulation resulted from STING trafficking to lysosomes. When bafilomycin A1 was used to block trafficking-mediated STING degradation,^17^ NeoL-induced abnormal lysosome distribution was indeed inhibited in Jurkat or Hut102 cells (Fig. 3F). We subsequently knocked out STING in Jurkat cells using CRISPR-Cas9 technology, which abolished STING expression and TBK1 phosphorylation (Fig. S7), and NeoL-induced lysosomal enlargement was also inhibited (Fig. 3G). Since TUBA1B mediated NeoL-induced STING trafficking, we also used shRNA to knock down *TUBA1B* and block STING trafficking, and NeoL-induced lysosomal enlargement was also suppressed (Fig. 3H).

To determine that NeoL-induced STING trafficking to lysosomes interferes with the function of NPC1 in removing cholesterol from lysosomes and resulting lysosomal cholesterol accumulation, we used atorvastatin to reduce cholesterol contents. In comparison to NeoL-treatment alone, the total expression of STING was lower in cells co-treated with atorvastatin and NeoL (Fig. 3I; Fig. S8–S10). This indicated that both cholesterol and STING occupied NPC1, and NeoL-induced STING trafficking blocked cholesterol transport. These results demonstrated that TUBA1B orchestrated NeoL-induced STING trafficking to lysosomes, and this disturbed the homeostasis of lysosomal cholesterol.

### Neoliensinine induces lysosomal disorders in T cell malignancies

#### *Neoliensinine promotes lysosomal damage in T cell malignancies*

**Figure 4:**
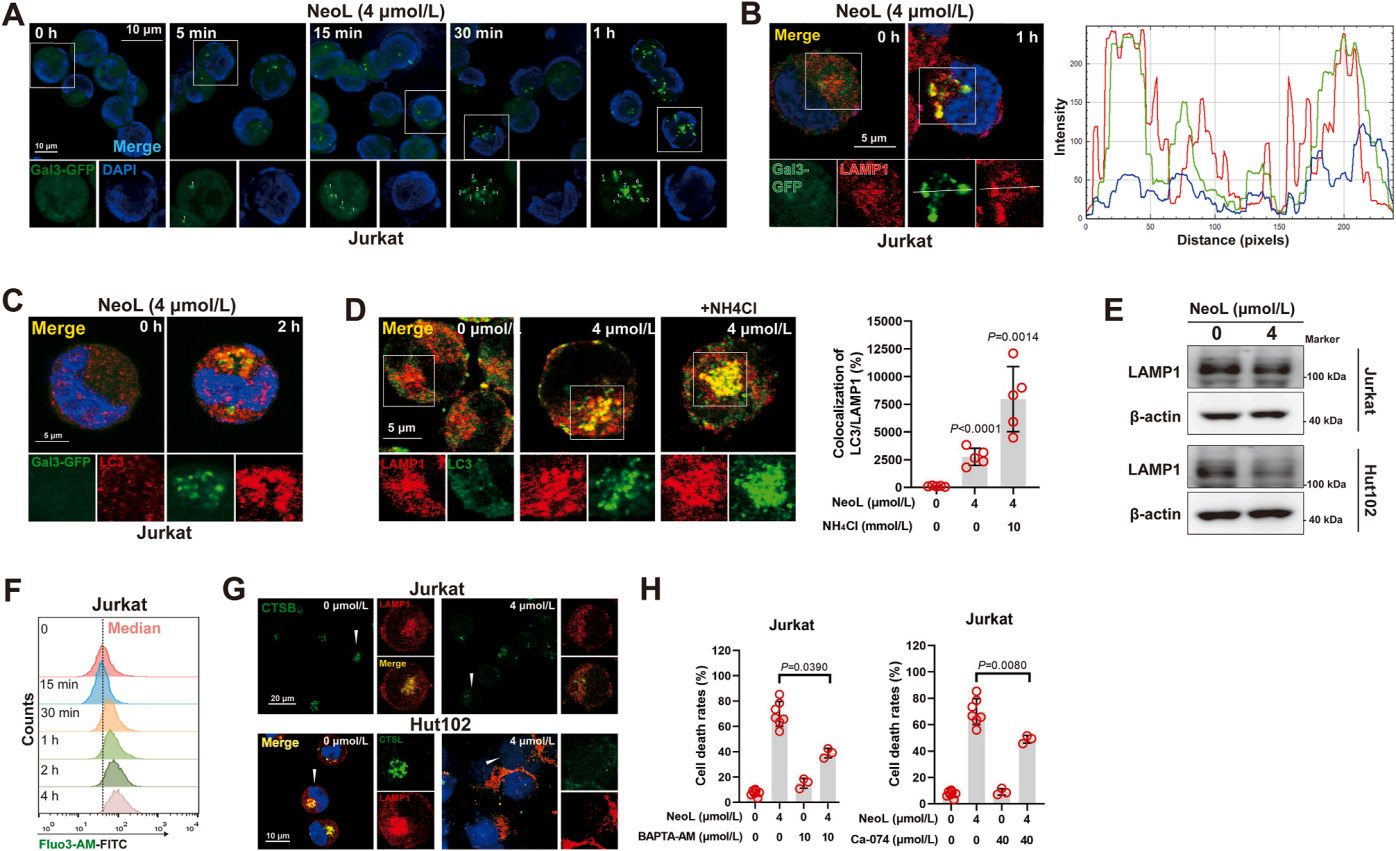
Neoliensinine promotes lysosomal disorders and lysosome-dependent cell death in T-cell malignancies. **(A)** The Jurkat cells stably expressing galectin-3-GFP (Gal3-GFP) were treated with 4 μmol/L of NeoL for 0–1 h, and imaged by confocal microscopy. Formation of Gal-3-GFP puncta was used as an indicator of lysosome damage, and Gal-3-GFP puncta were increased in a time-dependent manner after NeoL treatment. DAPI indicates the nucleus **(B)** Gal-3-GFP-expressing Jurkat cells were treated with 0 or 4 μmol/L of NeoL for 1 h, immunostained for LAMP1, and imaged using confocal microscopy. The coincidence of fluorescence was analyzed by Image J software. Gal3-GFP puncta were shown to co-localize with LAMP1 in lysosomes (RED) **(C)** Gal-3-GFP-expressing Jurkat cells were treated without or with 4 μmol/L of NeoL for 2 h, immunostained with anti-LC3 (Red) for autophagosomes, and imaged by confocal microscopy. NeoL treatment induced Gal3-GFP puncta and LC3 expression and their co-localization, suggesting that lysosomes damaged by NeoL were digested by autophagosomes **(D)** Jurkat cells were pre-treated with 10 mM NH_4_Cl (lysosomal alkalizer) for 1 h, and treated with 4 μmol/L of NeoL for 2 h, immunolabeled with LAMP1 (red) and LC3 (green), and imaged by confocal microscopy. Colocalization of LAMP1 and LC3 was quantified by Photoshop software. NeoL was shown to induce co-localization of LAMP1 and LC3, which were intensified by NH_4_Cl treatment **(E)** Jurkat and Hut102 cells were treated without or with 4 μmol/L of NeoL for 4 h, and the expression of LAMP1 was determined by Western blotting with β-actin as a loading control. NeoL was shown to reduce LAMP1 protein contents. **(F)** Jurkat cells were treated without or with 4 μmol/L of NeoL for 0–4 h. Cytoplasmic calcium contents were labeled with Fluo3-AM staining and quantified by flow cytometry. Calcium was shown to increase in a time-dependent manner after NeoL treatment. **(G)** Jurkat cells and Hut103 cells were treated with 0 or 4 μmol/L of NeoL for 2 h, and immunostained for LAMP1 and CTSB/CTSL. The enzymes were normally localized within the lysosome. Cells were imaged by confocal microscopy. Cells indicated by white arrowheads were shown with enlarged images on the right. The colocalization quantification of the two groups was shown in Figure S21. CTSB/CTSL were normally well-localized with LAMP1 in the lysosome. Under NeoL treatment, the CTSB/CTSL signals were widely dispersed in the cytoplasm, a sign of lysosome leakage or lysosomal membrane permeabilization (LMP). **(H)** The Jurka cells were pretreated without or with 10 μmol/L of BAPTA-AM (cytoplasmic calcium chelator) or 40 μmol/L of Ca-074 (a CTSB inhibitor) for 1 h, and then treated with 4 μmol/L of NeoL for 4 h. The cell death was determined by annexin V/PI staining (*n* ≥ 3). Both BAPTA-AM and Ca-074 were shown to partially reduce NeoL-induced Jurkat cell death, suggesting that both increased calcium contents and leakage of lysosomal enzymes contributed to NeoL-induced cell death. *P* values were indicated in the graph, and *P* < 0.05 indicates statistical significance.

NPC1 inhibition mediates cholesterol-associated lysosomal disorder, triggering cell death in cancer cells, including T-CMs.^26^^,^^28^ We then determined whether NeoL could induce the phenomenon of lysosomal disorders, including lysosomal damage, lysophagy (the repair mechanism when lysosomes are damaged,^29^ or lysosomal membrane permeabilization (LMP). Galectin-3 (Gal3) is recruited to lysosomes when the damage occurs, and Gal3 is a convenient and sensitive marker commonly used to detect lysosomal damage.^30^ T-CM cells were transfected with the Gal3-GFP-expressing plasmid, and weak green fluorescence puncta were observed in the cytoplasm of untreated cells (0 h; Fig. 4A), and Gal3 puncta formation was used to detect lysosomal damage.^31^ As shown in Figure 4A, NeoL quickly triggered the formation of Gal3-GFP puncta from 5 min of treatment. Within 1 h, both the ratio of lysosome-damaged cells and the number of puncta were increased sharply (Fig. S11). The Gal3-GFP puncta were formed on lysosomes after NeoL treatment, as they co-localized with LAMP1 (Fig. 4B). Thus, NeoL treatment indeed induced lysosomal damage.

#### *Lysophagy is evoked but blocked in T cell malignancies after neoliensinine treatment*

Damaged lysosomes can be triaged as a whole for degradation by autophagy (termed lysophagy). Ubiquitin chains can mark the damaged lysosomes, and then autophagy receptors (such as P62) induce and recruit the LC3-decorated phagophore for engulfment of the damaged lysosome for subsequent fusion with intact lysosomes for degradation.^29^ We then investigated NeoL-induced lysophagy in T-CMs by co-localization of Gal3-GFP puncta to mark damaged lysosomes and LC3 to mark autophagosomes (Fig. 4C). In NeoL-treated cells, the GFP puncta were highly colocalized with LC3 staining, suggesting that damaged lysosomes were engulfed by autophagosomes. Colocalization levels of lysosomes and autophagosomes were also enhanced in T-CMs after NeoL treatment, and the levels were further increased with lysosomal alkalizer (NH_4_Cl) (Fig. 4D), which was used to block autophagy flux and induce accumulation of undegraded substances. This was further supported by decreased LAMP1 in NeoL-treated cells, as a consequence of lysophagy (Fig. 4E).

Although lysophagy can clean up damaged lysosomes, the sustained lysosomal damage may result in autophagy blockage due to loss of lysosomal function.^26^ Firstly, un-obstructed autophagy flux can be defined by detecting degraded autophagy receptor.^32^ In NeoL-treated T-CMs cells, autophagy receptor (P62) was not markedly altered, although LC3II expression was significantly increased in a NeoL concentration- and time-dependent manner (Fig. S12, S13).

Secondly, we examined autophagy activity using T-CM cells stably transfected with a LC3-GFP-mCherry plasmid. Free autophagosomes appear as orange because both GFP and mCherry fluorescence are functional. However, when an autophagosome is fused with the lysosome, the GFP fluorescence will be quenched by acid pH condition in the lysosome, leaving mCherry fluorescence only. However, in NeoL-treated T-CMs cells, both red and green fluorescence puncta were well co-localized and increased in a time-dependent manner in cells transfected with LC3-GFP-mCherry, suggesting that NeoL blocked autophagy flux (Fig. S14).

#### *NeoL induced lysosomal membrane permeabilization and lysosome-dependent cell death in T cell malignancies*

Blocked autophagy flux suggested that NeoL resulted in damaged lysosome accumulation in T-CMs. Lysosome damage can be caused by LMP, which may induce cell death.^33^ LMP is accompanied by the leakage of lysosomal contents, such as cathepsins, calcium, and protons.^26^ When lysosomal acidity decreased, the intensity of fluorescence of LysoRED also declined, as LysoRED is acidic-dependent.^34^ Here, we determined whether LMP occurred in T-CMs after NeoL treatment. As shown in Figure 3, Figure 4 μmol/L of NeoL reduced lysosomal acidity after treatment for 1–4 h, suggesting proton leakage. NeoL enhanced cytoplasmic calcium levels in a time-dependent manner in three T-CM cell lines, indirectly suggesting the occurrence of LMP (Fig. 4F; Fig. S15).

Cathepsins are the lysosomal proteases, such as CTSB or CTSL, and their subcellular localization can be used as indicators of LMP occurrence.^35^ Under normal culture conditions, CTSB and CTSL staining (green; Fig. 4G) appeared in an aggregated form and were well co-localized with LAMP1 in lysosomes (red; Fig. 4G). However, under 4 μmol/L of NeoL treatment, colocalization of CTSB or CTSL with LAMP1 was reduced, and the green fluorescent signals of CTSB or CTSL were dispersed in the cytoplasm (Fig. 4G; Fig. S21). These data showed that NeoL did induce LMP in the T-CM cells.

We next investigated whether LMP was involved in NeoL-induced T-CM cell death. NeoL was shown to induce total cell death (necrosis, early and late apoptosis) in a concentration- and time-dependent manner (Fig. S16, S17), and 4 h of NeoL treatment at 4 μmol/L led to 69.86% ± 9.86%, 70.35% ± 17.74%, and 65.35% ± 14.47% cell death in Jurkat, Hut102, and Molt4 cells, respectively. Leakage of lysosomal contents by LMP could disrupt cellular homeostasis and survival, and BAPTA-AM, a cytoplasmic calcium chelator, or Ca-074, an inhibitor of CTSB, was indeed shown to attenuate NeoL-triggered cell death in three T-CM cell lines (Fig. 4H; Fig. S18), indicating that LMP contributed to NeoL-induced cell death. Therefore, NeoL-induced cell death of T-CMs is lysosome-dependent cell death.

### NeoL-induced lysosome disorders are mediated by STING-TUBA1B-lysosomal cholesterol axis

**Figure 5:**
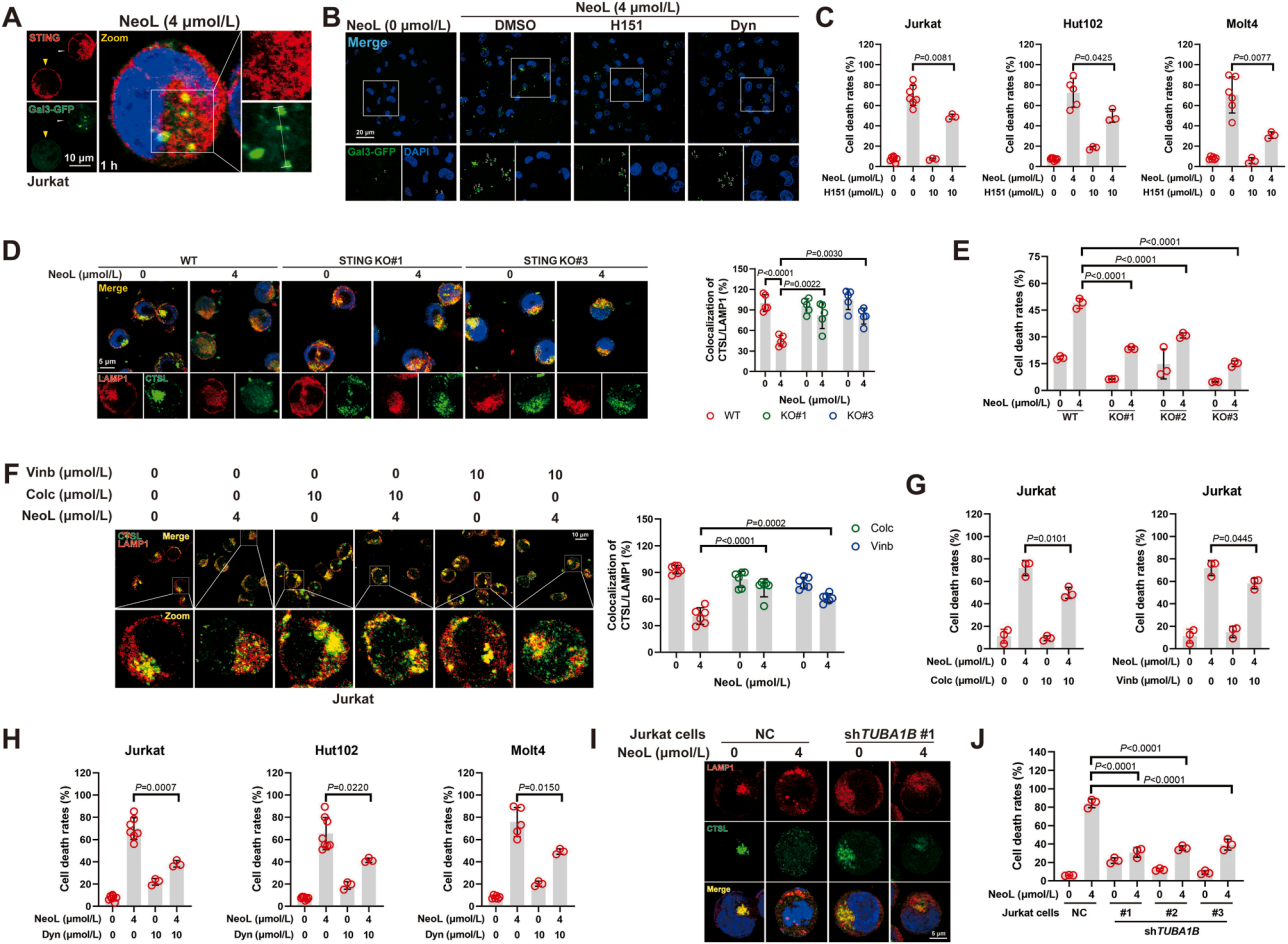
STING/TUBA1B mediates neoliensinine-induced lysosomal disorders and lysosome-dependent cell death in T-cell malignancies. **(A)** Gal3-GFP-expressing Jurkat cells were treated with 4 μmol/L of NeoL for 1 h, and immunolabeled for STING (red). Two cells in the same field under NeoL treatment were imaged by confocal microscopy. The white arrowhead indicates a cell with damaged lysosomes, and the yellow arrowhead shows a cell without obvious lysosome damage. The cell with lysosome damage was shown with an enlarged image to show co-localization of Gal3-GFP puncta with STING **(B)** Jurkat cells with the stable expression of Gal3-GFP were pretreated with 10 μmol/L of H151 (a STING inhibitor) or 10 μmol/L Dynasore (Dyn, a dynamin-dependent endocytosis blocker) for 1 h and then treated with 4 μmol/L of NeoL for 1 h, and imaged by confocal microscopy. The Gal3-GFP aggregates were labeled with 1–7 corresponding to increasing sizes of the puncta. Both the size and number of NeoL-induced Gal3-GFP puncta were reduced by H151 or Dyn treatment. **(C)** Jurkat, Hut102, or Molt4 cells were pre-treated with 10 μmol/L of H151 for 1 h and then treated with 4 μmol/L of NeoL for 4 h. The cell death rates were determined by annexin V/PI staining (*n* ≥ 3). The STING inhibitor was shown to significantly attenuate cell death in three T-cell malignancy cell lines **(D)** The lysosomal membrane permeabilization (LMP) phenomenon was determined by immunofluorescence assay and co-localization of CTSL and LAMP1. STING KO Jurkat cells (#1 and #3) were treated with 0 or 4 μmol/L of NeoL for 2 h. STING KO was shown to partially reverse NeoL's effect on CTSL distribution **(E)** STING KO Jurkat cells (#1, #2, and #3) were treated with 0 or 4 μmol/L of NeoL for 4 h. The cell death rates were determined by annexin V/PI staining (*n* = 3). NeoL-induced cell death was decreased in three STING KO Jurkat clones **(F)** Co-localization of CTSL and LAMP1 was used to determine LMP phenomena in Jurkat cells pre-treated with 10 μmol/L of microtubule destabilizers, colchicine (Colc) or vinblastine sulfate (Vinb), for 1 h and then treated with 0 or 4 μmol/L of NeoL for 2 h. Microtubule destabilizers were shown to ameliorate NeoL-induced lysosome leakage. **(G)** Jurkat cells were pre-treated for 1 h with 10 μmol/L of Colc or 10 μmol/L of Vinb, and then treated with 0 or 4 μmol/L NeoL for 4 h. The cell death rates were determined by annexin V/PI staining (*n* = 3). Both Colc and Vinb were shown to reduce NeoL-induced Jurkat cell death **(H)** Jurkat, Hut102, or Molt4 cells were pre-treated with 10 μmol/L of Dyn for 1 h and then treated with 4 μmol/L of NeoL for 4 h. The cell death rates were determined by annexin V/PI staining (*n* ≥ 3). Blocking dynamin-dependent lysosome endocytosis partially reduced NeoL-induced death rates in T-cell malignancy cells **(I)** The LMP phenomenon was determined by reduced co-localization of CTSL and LAMP1 immunofluorescence. Jurkat cells transfected with *TUBA1B* shRNA #1 showed improved co-localization of CTSL and LAMP1 under NeoL for 2 h. **(J)** Jurkat cells transfected with *TUBA1B* shRNA #1, #2, or #3 were treated with 0 or 4 μmol/L of NeoL for 4 h. The cell death was determined by annexin V/PI staining (*n* = 3). *TUBA1B* knockdown reduced NeoL-induced death of Jurkat cells. *P* values were indicated in the graph, and *P* < 0.05 indicates statistical significance.

Here, we explored the correlation between STING/TUBA1B axis-regulated lysosomal cholesterol accumulation and NeoL-induced lysosomal damage, LMP, and lysosome-dependent cell death. Jurkat cells were treated with 4 μmol/L NeoL for 1 h, and processed for immunofluorescence for the colocalization of STING and Gal3-GFP for damaged lysosomes. In the cell with lysosomal damage, STING was co-localized with Gal3-GFP partially (white arrow; Fig. 5A), while in the other cell, which had not yet experienced lysosomal damage, there were no Gal3-GFP puncta or colocalization with STING (yellow arrow; Fig. 5A; Fig. S19).

Next, we used H151, an STING inhibitor, and showed that H151 reduced the number and sizes of the Gal3-GFP puncta, and partially reversed NeoL-induced lysosomal damage (Fig. 5B; Fig. S20), LMP (Fig. S21), and cell death in T-CMs (Fig. 5C). Furthermore, in STING-knockout cells, NeoL-induced LMP was also attenuated, as CTSL was substantially co-localized with LAMP1 (Fig. 5D), and cell death was partially rescued (Fig. 5E).

Significant STING degradation only occurred when cells were treated with 4 μmol/L of NeoL (blue arrowhead; Fig. 1A). We now investigated if lower concentrations of NeoL could induce lysosomal damage. NeoL at 0.5, 1, or 2 μmol/L was also shown to induce accumulation of Gal3-GFP puncta, a sign of lysosomal damage (Fig. S22), indicating lysosomal damage is a more sensitive phenotype in NeoL-induced T-CM cell death, and NeoL-induced lysosome disorders were dependent on STING.

We then evaluated the correlation between microtubules and NeoL-induced lysosomal disorders. Treatment of microtubule destabilizers, colchicine or vinblastine sulfate, significantly inhibited NeoL-induced LMP (Fig. 5F) and cell death (Fig. 5G) in T-CMs. In addition, dynasore, a blocker of dynamin-dependent endocytosis which could delay the lysosomal trafficking of STING,^10^ was also shown to suppress the levels of NeoL-induced lysosomal damage (Fig. 5B; Fig. S20), LMP (Fig. 4G; Fig. S21), and cell death (Fig. 5H). Furthermore, *TUBA1B* knockdown also largely abolished NeoL-induced LMP (Fig. 5I) and cell death (Fig. 5J). These results demonstrated that microtubules, crucial for STING trafficking to lysosomes, played an important role in mediating NeoL-induced lysosome disorders.

**Figure 6:**
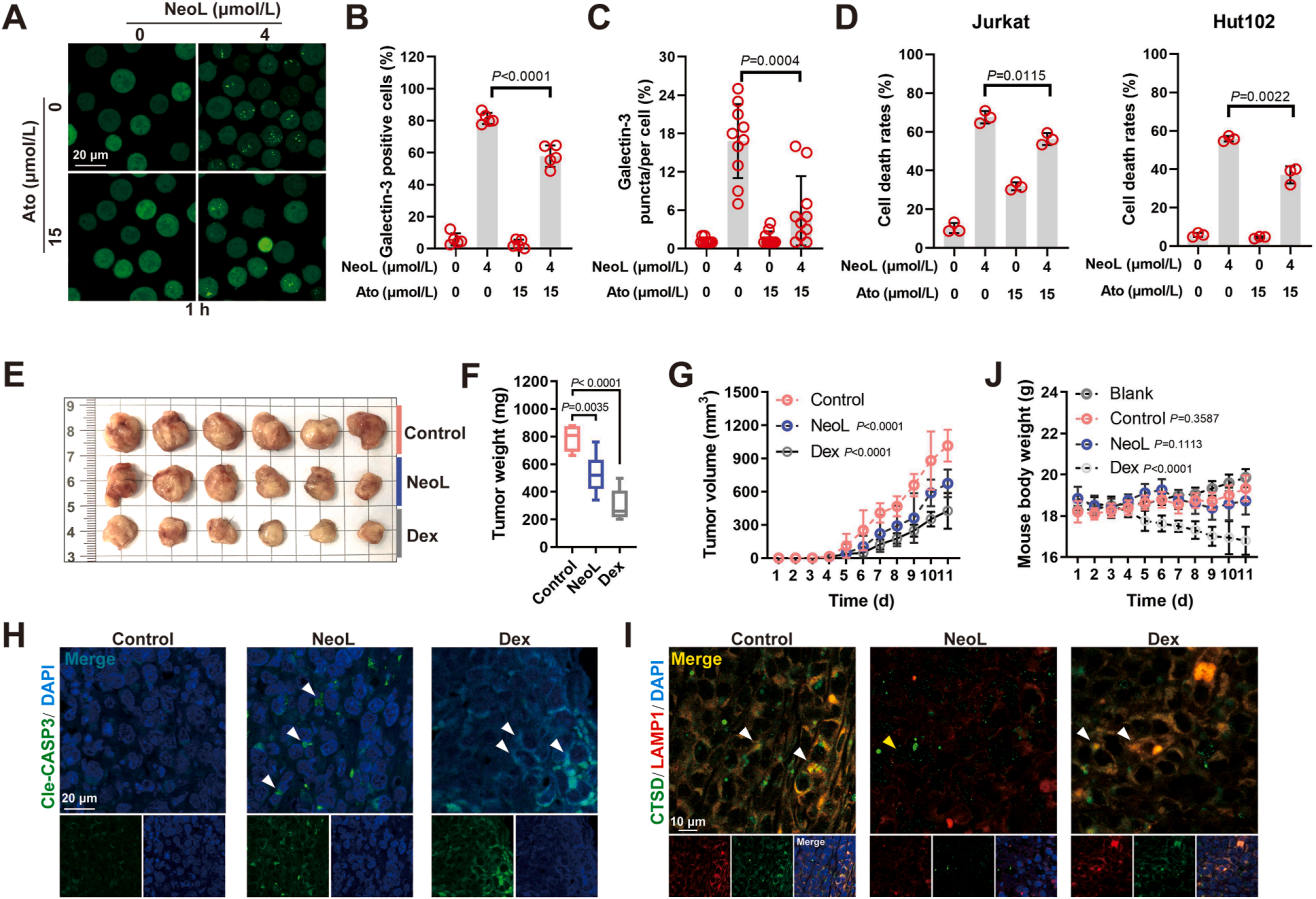
Cholesterol mediates neoliensinine-induced lysosomal disorder and neoliensinine inhibits T-cell malignancies *in vivo*. **(A**–**C)** Jurkat cells with the stable expression of Gal3-GFP were pre-treated with 0 or 15 μmol/L of atorvastatin (Ato, a cholesterol synthesis blocker) for 6 h and then treated with 0 or 4 μmol/L of NeoL for 1 h (A) Puncta of Gal3-GFP were determined by confocal microscopy. (B) The proportion of cells with Gal-3-GFP puncta and (C) the counts of Gal-3-GFP puncta in each cell were quantified. Ato treatment decreased both Gal-3-GFP puncta-positive cell number and Gal-3-GFP puncta/cells **(D)** Jurkat and Hut102 cells were pre-treated with 15 μmol/L of Ato for 6 h and then treated with 0 or 4 μmol/L of NeoL for 4 h. The cell death was determined by annexin V/PI staining (*n* = 3). Ato treatment improved T-cell malignancy cell survival under NeoL treatment. **(E, F)** The EL4 cells were inoculated into *C5*7BL*/6* mice (*n* = 6 for each group) for tumor formation for 11 days. (E) Tumors were recovered from mice treated with saline (top line), 5 mg/kg of NeoL (middle line), or 7.5 mg/kg dexamethasone (Dex; bottom line) daily for 11 days. (F) The tumor weight in each group was recorded and calculated at the terminal time point of the experiment. Both NeoL and Dex were shown to significantly reduce the tumor weight. **(G)** The tumor growth curve (volume) was recorded during the experiment. **(H, I)** Immunofluorescence analysis of sectioned tumors from each group. Cle-CASP3 indicates dead cells, LAMP1 indicates lysosomes, and CTSD indicates cathepsin D. **(J)** The body weight of mice was recorded during the experiment. In contrast to Dex, which declined body weight in a time-dependent manner from day 5, NeoL treatment did not show a significant effect on mouse body weight. *P* values were indicated in the graph, and *P* < 0.05 indicates statistical significance.

At last, we examined the correlation between cholesterol accumulation and lysosome disorders using cholesterol production inhibitor, atorvastatin. Pre-treatment with atorvastatin and inhibition of cholesterol production reduced NeoL-induced lysosomal damage, as Gal3-GFP puncta (Fig. 6A–C) and NeoL-induced cell death (Fig. 6D) were decreased, suggesting that cholesterol accumulation contributed to NeoL-induced lysosomal damage.

### Neoliensinine inhibited T cell malignancies *in vivo*

Prior to *in vivo* experiments, we next examined if NeoL exhibited any cytotoxicity to normal blood cells. PBMCs were recruited from heathy donors and cultured for 48 h with 0∼64 μmol/L of NeoL, and the inhibition rates of human PBMC viability were 7.87 ± 0.81 at 4 μmol/L, 8.20% ± 1.84% at 8 μmol/L, 8.85% ± 0.99% at 16 μmol/L, 17.80% ± 2.89% at 32 μmol/L, and 23.41% ± 1.89% at 64 μmol/L (Fig. S23), and the *IC*_50_ was 738.7 μmol/L at 48 h, grossly higher than *IC*_50_ 3.33 μmol/L at 12 h for T-CMs. For cell death after 24 h treatment, there was no significant change between 0 and 2–8 μmol/L of NeoL. The cell death rates of PBMCs after 24 h of NeoL treatment were 13.09% ± 5.86% at 0 μmol/L, 21.12% ± 3.67% at 2 μmol/L, 23.01% ± 3.28% at 4 μmol/L, and 22.71% ± 5.13% at 8 μmol/L (Fig. S24). These data showed that NeoL was substantially less toxic to normal PBMCs than to T-CM cells.

We subsequently investigated the *in vivo* efficiency of NeoL with a mouse T-CM cell line, EL4. 1 × 10^6^ EL4 cells were subcutaneously inoculated into the right leg of *C5*7BL*/6* mice, which were daily injected with saline, NeoL (5 mg/kg, MW 1028.3, 4.86 μmol/L), or dexamethasone (7.5 mg/kg, MW 392.5, 19.11 μmol/L) during 11 days of tumor growth. Mice treated with dexamethasone displayed 61.53% inhibition of tumor weight in comparison to those injected with saline. Mice that received 5 mg/kg of NeoL intraperitoneally showed inhibition of tumor weight by 32.8% (Fig. 6E and F) as well as the tumor volume (Fig. 6G). We simultaneously sectioned tumors from each group for immunofluorescence analysis. First, using cleaved-CASP3 to label dead cells, increased fluorescence signals were observed in the NeoL and dexamethasone groups, indicating that NeoL and dexamethasone induced cell death in tumor tissues (representative changes are indicated by white arrows) (Fig. 6H). Next, we also analyzed whether NeoL induced LMP in the subcutaneous tumors. The results showed that in the control and dexamethasone groups, LAMP1 and CTSD were co-localized (white arrows), whereas under NeoL treatment, CTSD became diffuse (yellow arrows) and its co-localization with LAMP1 was reduced (Fig. 6I), demonstrating that NeoL induced LMP occurrence *in vivo*.

Remarkably, NeoL showed no significant adverse effect on mouse body weight during experimentation (Fig. 6J), whereas dexamethasone significantly induced body weight loss, continuously from day 5 to day 11. We also histologically investigated the toxicity of NeoL (NeoL group) on the main organs of the mice, by comparing with normal *C5*7BL*/6* mice (blank group), EL4 cells-bearing mice (control group), and dexamethasone-treated mice (dexamethasone group) (Fig. S25). Compared with normal mice (blank group), obvious lesion was observed in the heart, kidney, brain, and lung tissues of both the control and NeoL groups. In liver tissues, NeoL alleviated EL4-induced nodules (green arrows) and hepatic sinus congestion (yellow arrows), and NeoL did not induce additional lesions in other main organs. There were no obvious lesions in the dexamethasone-treated group.

## Discussion

**Figure 7:**
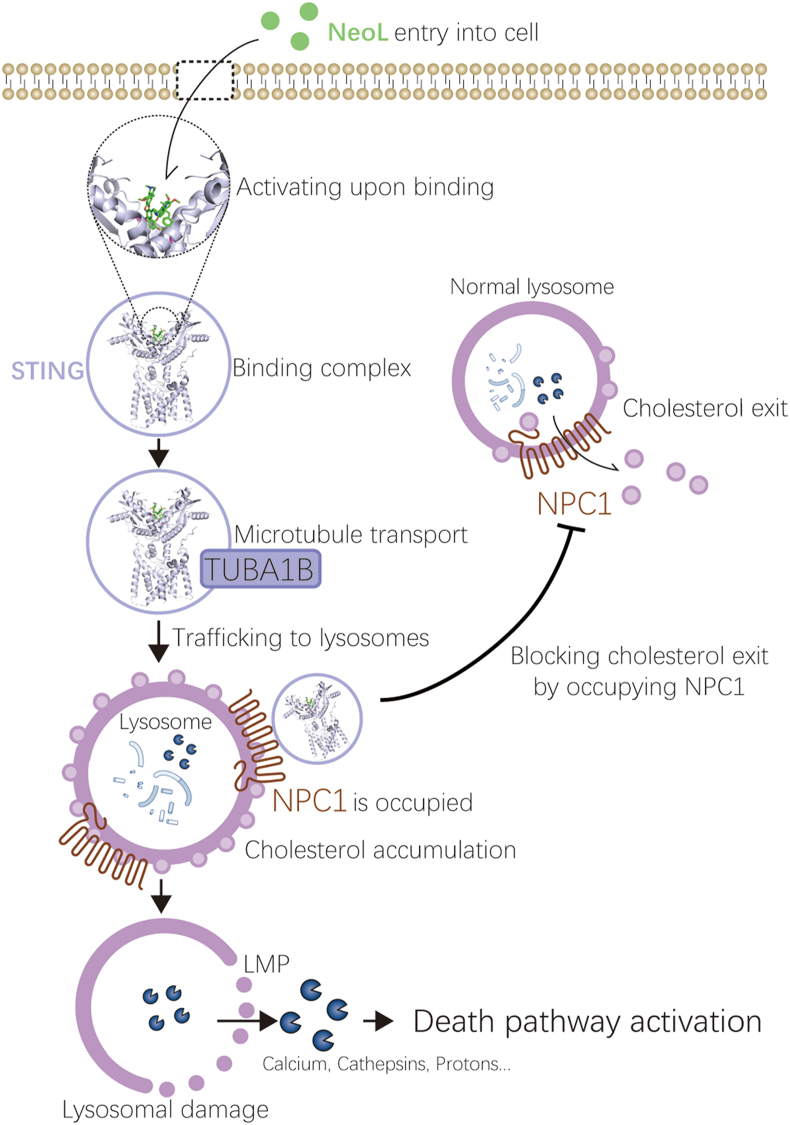
Mechanism diagram of NeoL-induced cell death in T-cell malignancies.

In this study, we reported the pro-cell death effects of NeoL, a natural product, on T-CM cells and uncovered a novel axis of STING-TUBA1B-cholesterol pathway mediating lysosome-dependent cell death (Fig. 7). We first identified the scarce tribenzylisoquinoline alkaloid, NeoL, from *Plumula nelumbinis*, which could inhibit the viability of T-CM cells with an *IC*_50_ of 3.33 μmol/L at 12 h, and showed more notable cytotoxicity than other bisbenzylisoquinoline alkaloids. We uncovered the mechanism by which NeoL-induced STING trafficking to lysosomes was mediated by TUBA1B, and illustrated that cholesterol was the inducer of STING-dependent lysosome-dependent cell death.

T-CMs are heterogeneous hematological malignancies with unmet medical needs. T-CMs include four major groups of peripheral T-cell lymphoma, cutaneous T-cell lymphomas, T-lymphocytic leukemia/lymphoma, and adult T-cell leukemia/lymphoma. Furthermore, the peripheral T-cell lymphoma group has over 30 subtypes, not otherwise specified, with angioimmunoblastic T-cell lymphoma and mesenchymal anaplastic large cell lymphoma as the common forms.^36^ The cutaneous T-cell lymphoma group specifically affects the skin, with mycosis fungoides and Sézary syndrome as the most common subtypes.^37^ The T-ALL/T-cell lymphoblastic lymphoma (T-LBL) group originates from T-cell precursors, manifesting either as T-ALL or T-LBL, whereas adult T-cell leukemia/lymphoma is caused by viral infection.

T-CMs are less well-understood and generally have a poorer prognosis compared with B-cell malignancies. Immunotherapy has currently been advanced with four FDA-approved chimeric antigen receptor-T cell products targeting CD19 for B-cell malignancies and with two targeting B-cell maturation antigens for multiple myeloma.^38^ Long-term outcomes of patients with B-cell lymphoma and/or chronic lymphocytic leukemia/small lymphocytic lymphoma receiving CD19-targeted chimeric antigen receptor-T cells demonstrated prolonged remissions in patients with B-cell malignancies. The overall response rates ranged from 44% to 91%, and complete response rates from 28% to 68% in 2–5 years of follow-up. Patients with Burkitt acute lymphoblastic leukemia showed a very high complete remission rate with CD19-targeted chimeric antigen receptor-T cells, exceeding 80% in many studies. Despite the adverse effects that exist, which include persistent B-cell/IgG depletion and infection, this is a remarkable progress towards the cure of B-cell malignancies.^38^

It is challenging to adopt current immunotherapy for T-CMs. This is largely because T-CMs are heterogeneous, and specific target antigens for their therapy are lacking. Over 17 proteins, including CD2, CD3, CD4, CD5, CD7, D26, CD30, CD37, CD47, CD70, CD276, CDC27, B7–H6, CCR4, CCR8, TCRVβ, and TRBC, are currently explored as therapeutic targets for T-CMs at preclinical and clinical stages.^2^ However, none is specific enough for T-CMs. This partially highlights the complexity in developing an effective immunotherapy for T-CMs.

The current treatment of T-CMs still relies largely on chemotherapy, followed by consolidation with autologous stem cell transplantation in the first remission.^39^ More specifically, the standard regimen for CD30-negative T-CMs is treated with CHOP (cyclophosphamide, doxorubicin, vincristine, and prednisone), or CHOEP (CHOP plus etoposide), as well as DHAP (dexamethasone, cytarabine, and cisplatin),^39^^,^^40^ whereas CD30-positive diseases are treated with CHP (cyclophosphamide, doxorubicin, and prednisone) plus brentuximab vedotin (an antibody (cAC10) against cell-membrane protein CD30). The use of the chemo-reagents are based on their nature to induce apoptosis of proliferative cells by incorporating into DNA (cyclophosphamide, cytarabine, cisplatin), disabling the DNA synthesis enzyme topoisomerase II (doxorubicin, etoposide), preventing tubulin polymerization and inducing mitotic crossing-over and higher frequency of aneuploid mitotic segregants (vincristine), or suppressing the immune system by binding to glucocorticoid receptors (prednisone-derived prednisolone, dexamethasone).

In the early stage of acute lymphoblastic leukemia treatment, glucocorticoids have been an important part of the treatment regimen for T-ALL,^41^ and the side effects are obvious.^42^ Despite the improvements in chemotherapeutic regimens, the therapeutic efficacy against most relapsed/refractory peripheral T-cell lymphoma subtypes and T-ALL/T-LBL is limited, cutaneous T-cell lymphomas have a variable prognosis, and the treatment of adult T-cell leukemia/lymphoma and natural killer/T-cell lymphoma remains challenging (Zhang et al, 2024). Nelarabine, another chemotherapeutic prodrug, only achieved an approximately 30% complete response rate in patients with relapsed/refractory T-ALL.^3^^,^^4^ In addition, the adverse effects of the chemotherapy are overwhelming, and the main side effect is toxicity to normal cells. The CD30 antibody (brentuximab vedotin) also received a black box warning with cases of progressive multifocal leukoencephalopathy, as CD30 is highly expressed in monocytes. It is therefore imperative to develop novel therapies to aid the unmet need of treatment for T-CMs.

In this research, we identified a natural compound, NeoL, from *Plumula nelumbinis*. It is worth noting that T-CMs were far more sensitive to NeoL-induced cell death than normal PBMCs. An effective T-CM killing in culture was demonstrated with micromolar concentration of NeoL overnight, and a moderate but significant inhibition of tumor growth was also detected in the pre-clinical investigation. STING is known to induce cell death in tumor cells, and we detected a high level of STING expression in the T-CMs. NeoL was shown to activate STING at 1 or 2 μmol/L concentration but promote STING degradation at 4 μmol/L concentration. Until now, not much information is available on how STING degrades. A previous report showed that STING could be transported to lysosomes,^10^ and we indeed detected localization of STING in lysosomes after NeoL treatment. This might be because highly expressed STING was more frequently transported to lysosomes for degradation after activation, preventing STING from hyperactivating the response that produces immune dysregulation.^43^

The microtubules have been recently recognized to mediate STING transportation and degradation.^44^, ^45^, ^46^ However, how STING travels to lysosomes is currently unknown. In this report, we identified TUBA1B, a novel molecule that could mediate NeoL-induced STING trafficking to lysosomes. TUBA1B is a microtubule isoform involved in the formation of the cytoskeleton and is identified in anti-STING precipitates by liquid chromatography/mass spectrometry, thus establishing a direct interaction between TUBA1B and STING. Our results further showed that microtubule destabilizers suppressed NeoL-induced STING degradation in T-CMs, and *TUBA1B* knockdown attenuated the whole series of NeoL-induced effects, including STING-LAMP1 colocalization, lysosomal damage, LMP, and cell death. This finding explained the pivotal protein of STING transport to lysosomes under the treatment of NeoL, which is crucial for revealing the death mechanism of STING-induced lysosome-dependent cell death. The auxiliary role of TUBA1B is to provide the necessary conditions for STING transport and induction of cell death. We agree with another report that degradation of activated STING may be a protective mechanism for cells, organs, or the body to avoid an over-immune response under normal circumstances.^43^ Therefore, in the condition of enhancing immune response, cutting off STING lysosomal transport by inhibiting TUBA1B may contribute stronger immune response and avoid STING-induced cell death.

The mechanism of STING trafficking to lysosome-induced LMP remains unclear. In NeoL-treated T-CM cells, accumulation of cholesterol in lysosomes was observed. Our previous studies, along with others, regarded lysosomal cholesterol accumulation as a trigger for lysosomal damage and subsequent LMP.^26^^,^^47^ The classic function of NPC1 is to export cholesterol from lysosomes.^24^ Occupation of NPC1 can therefore suppress cholesterol export from lysosomes.^25^ One study adds to the evidence linking STING and cholesterol by demonstrating that NPC1 interacts with STING and recruits it to lysosomes for degradation.^23^ Our study proved the effects of cholesterol and STING on NeoL-induced lysosomal damage, especially, the elimination of cholesterol could accelerate the degradation of STING under NeoL treatment. Based on these findings, we proposed a novel STING-TUBA1B-cholesterol axis in which STING degradation occupies NPC1 and limits cholesterol export, resulting in cholesterol accumulation in lysosomes and lysosomal disorders.

The current immunotherapy still needs time to overcome many obstacles to develop treatment for T-CMs, and innovation of traditional therapy is still necessary, because it can provide a timely response to T-CM patients who need treatment. A recent study demonstrated the possibility of screening sensitive drugs for the treatment of specific cancer cells in specific patients.^7^ The results of our study on NeoL are effective in T-CMs that have high expression of STING, and STING also needs to be rapidly activated before it can be transported to lysosomes to induce cell death.

In comparison to the effects achieved with 4 μmol/L of NeoL in 2 h, the existing STING agonist (2′,3′-cGAMP) requires a much higher concentration (20 μmol/L) and longer action time (12 h), even though it did not cause STING degradation. Therefore, to achieve effective STING-induced lysosome-dependent cell death, it requires a fast and strong process of STING regulation is required. Therefore, if tumor cells of clinical T-CM patients can be screened for the characteristics of high STING expression, NeoL may be tested for therapeutic effects in clinical settings.

In our study, we demonstrated that NeoL could be safe to treat T-CMs both *in vivo* and *in vitro*. First, no significant toxicity to PBMCs was observed over a longer duration of action than T-CMs under much higher concentrations. Secondly, the *in vivo* experiments showed that, different from dexamethasone, NeoL demonstrated no obvious effect on body weight. In addition, no pathological phenotype was detected among mouse organs after 11 days of NeoL treatment. The tumor selectivity of NeoL may be due to the dependence of tumor cells on lysosomes. Cancer cells rely on lysosome-dependent degradation to recycle nutrients that serve their energetic and biosynthetic needs.^48^ Tumor cells are more dependent on lysosomes to survive, so they are also more likely to respond to drug-induced lysosome-dependent cell death.

We propose that NeoL-induced T-CM death may be combined with chemotherapy, and this may further improve the therapeutic outcome of T-CMs. One of the advantages of NeoL-combined chemotherapy is that whereas chemotherapy causes protective autophagy, NeoL can inhibit lysosome and autophagy pathways by inducing lysosome-dependent cell death, thus inhibiting the self-rescue pathway of tumor cells, which may be investigated in the follow-up studies.^49^^,^^50^

We identified the natural compound NeoL and showed that NeoL induced cell death in culture and reduced tumor growth in mice bearing T-CM cells. We also identified TUBA1B mediating STING trafficking to lysosomes for degradation, which disturbed lysosomal cholesterol homeostasis and induced lysosomal disorders. The study also uncovered the link between STING and lysosome-dependent cell death. NeoL was shown to be safe and reduce tumor growth in mice with T-CMs. These results not only enhance our understanding of STING's role but also position NeoL as a promising therapeutic agent to improve the treatment of T-CMs.

## CRediT authorship contribution statement

**Po Hu:** Investigation, Data curation, Funding acquisition, Methodology, Conceptualization, Visualization, Formal analysis, Writing – original draft, Writing – review & editing. **Yuxuan Huang:** Investigation. **Zipeng Yu:** Investigation. **Xiaolong Zhang:** Investigation. **Guangming Yang:** Writing – original draft, Conceptualization, Formal analysis, Methodology, Funding acquisition. **Yang Pan:** Methodology, Conceptualization, Writing – original draft, Formal analysis.

## Data availability

All data are included in the main text or Supplementary materials. All data generated or analyzed during this study are included in this published article and its Supplementary files.

## Ethics declaration

All experimental protocols of animals were strictly conformed to the guidelines for the Guide of the Care and Use of Laboratory Animals and were approved by the Ethical Committee of Nanjing University of Chinese Medicine (202412A009). The experiments using human samples were approved by the IRB of the Affiliated Hospital of Nanjing University of Chinese Medicine (2022NL-KS006).

## Funding

This work was supported by the 10.13039/501100001809National Natural Science Foundation of China (No. 82204420, 82474123), the 10.13039/501100004608Natural Science Foundation of Jiangsu Province, China (No. BK20220474), the Funding Supporting Projects of 10.13039/501100001809National Natural Science Foundation of China (No. XPT82204420), the National Excellent Youth Cultivation Project of Nanjing University of Chinese Medicine (China) (No. RC202408), the Chinese Medicine First-Class Scientific Research and Cultivation Project of Nanjing University of Chinese Medicine (China) (No. ZYXPY2024-007, ZYXYL2024-006), and the 2023 Jiangsu Science and Technology Association Youth Science and Technology Talent Lifting Project (China) (No. TJ-2023-060).

## Conflict of interests

The authors declared no conflict of interests.
