# Supplementary material for: Neoliensinine promotes lysosomal cell death in T cell malignancies via a novel STING-TUBA1B pathway
Source: Genes Dis. 2025 Aug 14;13(6):101809. doi: 10.1016/j.gendis.2025.101809 (PMC13485499; doi:10.1016/j.gendis.2025.101809)
Supplement: Supplementary file 1 [file mmc1.docx]

**Supplementary Figures**

Supplementary Figure 1. The expression of STING in untreated cell lines of T-cell malignancies (T-CMs) and B-cell malignancies (B-CMs) was determined by western blot. β-actin was used as loading control.

Supplementary Figure 2. Jurkat cells were treated with 20 μmol/L of 2',3'-cGAMP for 0 - 24 h, and the expression of p-TBK1, p-STING and STING was determined by western blot. β-actin was used as a loading control. The quantification of p-STING and STING were performed.

Supplementary Figure 3. The expression of *STING* mRNA in Jurkat or Hut102 cells pre-treated with 10 μmol/L of Colc or 10 μmol/L of Vinb for 1 h and then treated with 4 μmol/L of NeoL for 4 h (*n* = 3). The *P* value was indicated in the graph, and *P* < 0.05 indicates the significance.

Supplementary Figure 4. Jurkat cells transfected with *TUBA1B* shRNA #1 were treated with 4 μmol/L of NeoL for 15 min. The lysosomal enlargement was determined by lysotracker RED (*n* = 5). The *P* value was indicated in the graph, and *P* < 0.05 indicates the significance.

Supplementary Figure 5. Quantification of Figure 2F (*n* = 3). The *P* value was indicated in the graph, and *P* < 0.05 indicates the significance.

Supplementary Figure 6. Hut102 cells were pre-treated with 15 μmol/L of atorvastatin (Ato) for 6 h and then treated with 4 μmol/L of NeoL for 4 h. Cells stained with lysotracker RED were determined by flow cytometry (*n* = 3). The *P* value was indicated in the graph, and *P* < 0.05 indicates the significance.

Supplementary Figure 7. The STING expression was knockout by Crispr-Cas9 in Jurkat cells. The expression of STING and p-TBK1 was determined by western blot. HSP70 was used as a loading control.

Supplementary Figure 8. Hut102 cells were pre-treated with 15 μmol/L of Ato for 6 h and then treated with 4 μmol/L of NeoL for 4 h. Expression of p-STING, STING and p-TBK1 was determined by western blot. β-actin was used as a loading control.

Supplementary Figure 9. Quantification of Supplementary Figure 8 (*n* = 3). The *P* value was indicated in the graph, and *P* < 0.05 indicates the significance.

Supplementary Figure 10. Quantification of Figure 3I (*n* = 3). The *P* value was indicated in the graph, and *P* < 0.05 indicates the significance.

Supplementary Figure 11. The number of cells with Gal-3-GFP puncta (left) and counts of Gal-3-GFP puncta in each cell (right) were calculated from Figure 4A (*n* ≥ 3).

Supplementary Figure 12. Jurkat cells were treated with 4 μmol/L of NeoL for 0 - 4 h, and the expression of LC3 and P62 was determined by western blot. β-actin was used as a loading control (*n* = 3). The *P* value was indicated in the graph, and *P* < 0.05 indicates the significance.

Supplementary Figure 13. Hut102 cells were treated with 0 - 4 μmol/L of NeoL for 4 h, and the expression of LC3 and P62 was determined by western blot. β-actin was used as a loading control (*n* = 3). The *P* value was indicated in the graph, and *P* < 0.05 indicates the significance.

Supplementary Figure 14. Jurkat cells-expressing LC3-GFP-mcherry stably were treated with 4 μmol/L of NeoL for 0 - 4 h, the formation of LC3-GFP-mcherry puncta was determined by confocal microscopy. The LC3-GFP-mcherry puncta were calculated (*n* ≥ 4). *P* < 0.05 indicates the significance.

Supplementary Figure 15. The cytoplasmic concentration of calcium in Hut102 and Molt4 cells treated with 4 μmol/L of NeoL for 0 - 6 h were detected by Fluo3-AM staining and flow cytometer.

Supplementary Figure 16. Jurkat were treated with 0 - 8 μmol/L of NeoL for 4 h. Hut102 cells were treated with 0 - 4 μmol/L of NeoL for 4 h. Molt4 cells were treated with 0 or 4 μmol/L of NeoL for 4 h. Cell death were detected by Annexin V/PI staining. Annexin V^+^/PI^-^: early apoptosis; Annexin V^+^/PI^+^: late apoptosis; Annexin V^-^/PI^+^: necrosis; total cell death: sum values other than the quadrant of Annexin V^-^/PI^-^ (the same as in the following contents) (*n* ≥ 3). The *P* value was indicated in the graph, and *P* < 0.05 indicates the significance.

Supplementary Figure 17. T-CMs cell lines were treated with 4 μmol/L of NeoL for 0 – 6/8 h. Total cell death were detected by Annexin V/PI staining (*n* ≥ 3). The *P* value was indicated in the graph, and *P* < 0.05 indicates the significance.

Supplementary Figure 18. The Hut102 and Molt4 cells were pretreated with 10 μmol/L of BAPTA-AM or 40 μmol/L of Ca-074 for 1 h, and then treated with 4 μmol/L of NeoL for 4 h. The cell death was determined by Annexin V/PI staining (*n* ≥ 3). The *P* value was indicated in the graph, and *P* < 0.05 indicates the significance.

Supplementary Figure 19. The coincidence of fluorescence of STNG (red) and Gal3-GFP (green) was analyzed from Figure 5A by Image J software.

Supplementary Figure 20. The counts of Gal-3-GFP puncta in each cell (left) and number of cells with Gal-3-GFP puncta (right) were calculated from Figure 5B (*n* ≥ 3).

Supplementary Figure 21. The LMP phenomena (co-localization of CTSB/CTSL and LAMP1) was determined by immunofluorescence assay in Jurkat or Hut102 cells pre-treated with 10 μmol/L of H151 or 10 μmol/L of Dyn for 1 h and then treated with 0, 4 μmol/L of NeoL for 2 h. The colocalization quantification shown in right was combined the groups in Figure 4G (*n* ≥ 3). Cell indicated by white arrowhead was shown with magnified image underneath.

Supplementary Figure 22. The Jurkat cells with the stable expression of Galectin-3-GFP (Gal3-GFP) were treated with 0, 0.5, 1, and 2 μmol/L of NeoL for 4 h and the formation of Gal-3-GFP puncta were detected by confocal microscopy. DAPI indicates nucleus.

Supplementary Figure 23. The cell viability was determined by MTT assay in PBMCs that treated with 0-64 μmol/L of NeoL for 48 h.

Supplementary Figure 24. PBMCs were treated with 0-8 μmol/L of NeoL for 24 h. Total cell death were detected by Annexin V/PI staining (*n* ≥ 3). The *P* value was indicated in the graph, and *P* < 0.05 indicates the significance.

Supplementary Figure 25. H&E staining of mouse organs for pathological evaluation of EL4-bearing mice treated with NeoL or Dex.
