# Supplementary material for: Neoliensinine promotes lysosomal cell death in T cell malignancies via a novel STING-TUBA1B pathway
Source: Genes Dis. 2025 Aug 14;13(6):101809. doi: 10.1016/j.gendis.2025.101809 (PMC13485499; doi:10.1016/j.gendis.2025.101809)
Supplement: Supplementary file 2 [file mmc2.docx]

**Supplementary Tables**

**Supplementary Table 1 STING-CoIP-LC-MS/MS of Jurkat cells treated with 2', 3'-cGAMP**

| **No.** | **Score Sequest HT** | **Gene Symbol** | **Cellular Component** | **Abundances** |
| --- | --- | --- | --- | --- |
| 1 | 218.63 | KRT1 | non-structural extracellular;plasma membrane;other membranes;cytosol;cytoskeleton;other cell component | 3202963129 |
| 2 | 203.8 | KRT2 | plasma membrane;cytosol;cytoskeleton;other cell component | 3005094466 |
| 3 | 160.27 | KRT10 | plasma membrane;cytosol;cytoskeleton;other cell component | 3456169975 |
| 4 | 84.44 | KRT9 | cytosol;cytoskeleton | 769047387.6 |
| 5 | 101.58 | KRT5 | cytosol;cytoskeleton;other cell component | 192259288.1 |
| 6 | 79.51 | KRT14 | plasma membrane;cytosol;cytoskeleton;nucleus;other cell component | 184554064.6 |
| 7 | 82.67 | KRT6A | cytosol;cytoskeleton | 39096391.81 |
| 8 | 50.93 | RPL7 | cytosol;translational apparatus;other cell component | 1008749038 |
| 9 | 72.24 | KRT16 | plasma membrane;cytosol;cytoskeleton | 12843372.63 |
| 10 | 39.09 | KRT17 | plasma membrane;cytosol;cytoskeleton | 7494916 |
| 11 | 31.72 | RPL35 | cytosol;translational apparatus;nucleus;other cell component | 342853521 |
| 12 | 23.65 | **TUBA1A** | plasma membrane;other membranes;cytosol;cytoskeleton;other cytoplasmic organelle;other cell component | 45903542.69 |
| 13 | 34.99 | RPL18 | cytosol;ER/Golgi;translational apparatus;nucleus;other cell component | 200530213.9 |
| 14 | 14.48 | TUBB | non-structural extracellular;other membranes;cytosol;cytoskeleton;nucleus;other cytoplasmic organelle;other cell component | 35731787.81 |
| 15 | 14.08 | YBX1 | non-structural extracellular;plasma membrane;other membranes;cytosol;ER/Golgi;nucleus;other cell component | 30982515.5 |
| 16 | 20.92 | RPL19 | cytosol;translational apparatus;nucleus;other cell component | 129522940.1 |
| 17 | 15.44 | EEF1A1 | non-structural extracellular;plasma membrane;other membranes;cytosol;cytoskeleton;translational apparatus;nucleus;other cytoplasmic organelle;other cell component | 63874769 |
| 18 | 22.68 | H4C1; H4C11; H4C12; H4C13; H4C14; H4C15; H4C16; H4C2; H4C3; H4C4; H4C5; H4C6; H4C8; H4C9 | non-structural extracellular;nucleus;other cell component | 155634565.3 |
| 19 | 9.63 | SURF6 | nucleus;other cell component | 67751640.06 |
| 20 | 13.96 | ACTB | plasma membrane;other membranes;cytosol;cytoskeleton;nucleus;other cell component | 42723385.25 |

**Supplementary Table 2 STING-CoIP-LC-MS/MS of Jurkat cells treated with NeoL**

| **No.** | **Score Sequest HT** | **Gene Symbol** | **Cellular Component** | **Abundances** |
| --- | --- | --- | --- | --- |
| 1 | 159.02 | KRT1 | non-structural extracellular;plasma membrane;other membranes;cytosol;cytoskeleton;other cell component | 2035168773 |
| 2 | 138.63 | KRT2 | plasma membrane;cytosol;cytoskeleton;other cell component | 886828404.2 |
| 3 | 130.22 | KRT10 | plasma membrane;cytosol;cytoskeleton;other cell component | 1849474434 |
| 4 | 63.08 | KRT9 | cytosol;cytoskeleton | 434049951.8 |
| 5 | 64.26 | RPL4 | cytosol;ER/Golgi;translational apparatus;nucleus;other cell component | 440301375.8 |
| 6 | 58.23 | RPL7 | cytosol;translational apparatus;other cell component | 839708609.6 |
| 7 | 63.98 | KRT5 | cytosol;cytoskeleton;other cell component | 90825545.69 |
| 8 | 49.47 | KRT14 | plasma membrane;cytosol;cytoskeleton;nucleus;other cell component | 89324589 |
| 9 | 51.54 | KRT6A | cytosol;cytoskeleton | 19330884 |
| 10 | 55.69 | RPL18 | cytosol;ER/Golgi;translational apparatus;nucleus;other cell component | 698354483.3 |
| 11 | 25.37 | TUBB | non-structural extracellular;other membranes;cytosol;cytoskeleton;nucleus;other cytoplasmic organelle;other cell component | 35582913.59 |
| 12 | 39.75 | KRT16 | plasma membrane;cytosol;cytoskeleton | 1750141 |
| 13 | 39.31 | ALB | non-structural extracellular;ER/Golgi;other cell component | 1291184099 |
| 14 | 21.13 | ACTB | plasma membrane;other membranes;cytosol;cytoskeleton;nucleus;other cell component | 48567676.38 |
| 15 | 16.51 | **TUBA1B** | cytoskeleton;other cell component | 38115838.13 |
| 16 | 26.96 | RPS6 | cytosol;ER/Golgi;translational apparatus;nucleus;other cell component | 91240987.75 |
| 17 | 23.54 | RPL15 | cytosol;translational apparatus;nucleus;other cell component | 81497705.75 |
| 18 | 30 | EEF1A1 | non-structural extracellular;plasma membrane;other membranes;cytosol;cytoskeleton;translational apparatus;nucleus;other cytoplasmic organelle;other cell component | 72646089.25 |
| 19 | 15.74 | HSP90AB1 | non-structural extracellular;plasma membrane;other membranes;cytosol;nucleus;other cell component | 30323646.06 |
| 20 | 19.25 | HSPA8 | non-structural extracellular;plasma membrane;other membranes;cytosol;nucleus;other cytoplasmic organelle;other cell component | 25219134.56 |

**Supplementary Table 3 ESCRT-related proteins listed in Uniprot (**[**https://www.uniprot.org/**](https://www.uniprot.org/)**).**

| **Entry** | **Reviewed** | **Entry Name** | **Protein names** | **Gene Names** | **Organism** | **Length** |
| --- | --- | --- | --- | --- | --- | --- |
| A0A2R8Y4I8 | unreviewed | A0A2R8Y4I8_HUMAN | Spastin (EC 5.6.1.1) | SPAST SPG4 | Homo sapiens (Human) | 583 |
| A0A2U3TZR0 | unreviewed | A0A2U3TZR0_HUMAN | Spastin (EC 5.6.1.1) | SPAST SPG4 | Homo sapiens (Human) | 615 |
| A5D8V6 | reviewed | VP37C_HUMAN | Vacuolar protein sorting-associated protein 37C (hVps37C) (ESCRT-I complex subunit VPS37C) | VPS37C PML39 | Homo sapiens (Human) | 355 |
| A6NHL2 | reviewed | TBAL3_HUMAN | Tubulin alpha chain-like 3 (EC 3.6.5.-) | TUBAL3 | Homo sapiens (Human) | 446 |
| A6NNZ2 | reviewed | TBB8B_HUMAN | Tubulin beta 8B | TUBB8B | Homo sapiens (Human) | 444 |
| E5KRP5 | unreviewed | E5KRP5_HUMAN | Spastin (EC 5.6.1.1) | SPAST SPG4 hCG_23080 | Homo sapiens (Human) | 616 |
| E5KRP6 | unreviewed | E5KRP6_HUMAN | Spastin (EC 5.6.1.1) | SPAST SPG4 | Homo sapiens (Human) | 584 |
| O00308 | reviewed | WWP2_HUMAN | NEDD4-like E3 ubiquitin-protein ligase WWP2 (EC 2.3.2.26) (Atrophin-1-interacting protein 2) (AIP2) (HECT-type E3 ubiquitin transferase WWP2) (WW domain-containing protein 2) | WWP2 | Homo sapiens (Human) | 870 |
| O14559 | reviewed | RHG33_HUMAN | Rho GTPase-activating protein 33 (Rho-type GTPase-activating protein 33) (Sorting nexin-26) (Tc10/CDC42 GTPase-activating protein) | ARHGAP33 SNX26 TCGAP | Homo sapiens (Human) | 1287 |
| O14964 | reviewed | HGS_HUMAN | Hepatocyte growth factor-regulated tyrosine kinase substrate (Hrs) (Protein pp110) | HGS HRS | Homo sapiens (Human) | 777 |
| O15455 | reviewed | TLR3_HUMAN | Toll-like receptor 3 (CD antigen CD283) | TLR3 | Homo sapiens (Human) | 904 |
| O43633 | reviewed | CHM2A_HUMAN | Charged multivesicular body protein 2a (Chromatin-modifying protein 2a) (CHMP2a) (Putative breast adenocarcinoma marker BC-2) (Vacuolar protein sorting-associated protein 2-1) (Vps2-1) (hVps2-1) | CHMP2A BC2 CHMP2 | Homo sapiens (Human) | 222 |
| O60493 | reviewed | SNX3_HUMAN | Sorting nexin-3 (Protein SDP3) | SNX3 | Homo sapiens (Human) | 162 |
| O60749 | reviewed | SNX2_HUMAN | Sorting nexin-2 (Transformation-related gene 9 protein) (TRG-9) | SNX2 TRG9 | Homo sapiens (Human) | 519 |
| O75340 | reviewed | PDCD6_HUMAN | Programmed cell death protein 6 (Apoptosis-linked gene 2 protein homolog) (ALG-2) | PDCD6 ALG2 | Homo sapiens (Human) | 191 |
| O75351 | reviewed | VPS4B_HUMAN | Vacuolar protein sorting-associated protein 4B (EC 3.6.4.6) (Cell migration-inducing gene 1 protein) (Suppressor of K(+) transport growth defect 1) (Protein SKD1) | VPS4B SKD1 VPS42 MIG1 | Homo sapiens (Human) | 444 |
| O75382 | reviewed | TRIM3_HUMAN | Tripartite motif-containing protein 3 (EC 2.3.2.27) (Brain-expressed RING finger protein) (RING finger protein 22) (RING finger protein 97) | TRIM3 BERP RNF22 RNF97 | Homo sapiens (Human) | 744 |
| O75531 | reviewed | BAF_HUMAN | Barrier-to-autointegration factor (Breakpoint cluster region protein 1) [Cleaved into: Barrier-to-autointegration factor, N-terminally processed] | BANF1 BAF BCRG1 | Homo sapiens (Human) | 89 |
| O75843 | reviewed | AP1G2_HUMAN | AP-1 complex subunit gamma-like 2 (Gamma2-adaptin) (G2ad) | AP1G2 | Homo sapiens (Human) | 785 |
| O75886 | reviewed | STAM2_HUMAN | Signal transducing adapter molecule 2 (STAM-2) (Hrs-binding protein) | STAM2 HBP | Homo sapiens (Human) | 525 |
| O95219 | reviewed | SNX4_HUMAN | Sorting nexin-4 | SNX4 | Homo sapiens (Human) | 450 |
| O95630 | reviewed | STABP_HUMAN | STAM-binding protein (EC 3.4.19.-) (Associated molecule with the SH3 domain of STAM) (Endosome-associated ubiquitin isopeptidase) | STAMBP AMSH | Homo sapiens (Human) | 424 |
| P00533 | reviewed | EGFR_HUMAN | Epidermal growth factor receptor (EC 2.7.10.1) (Proto-oncogene c-ErbB-1) (Receptor tyrosine-protein kinase erbB-1) | EGFR ERBB ERBB1 HER1 | Homo sapiens (Human) | 1210 |
| P04350 | reviewed | TBB4A_HUMAN | Tubulin beta-4A chain (Tubulin 5 beta) (Tubulin beta-4 chain) | TUBB4A TUBB4 TUBB5 | Homo sapiens (Human) | 444 |
| P08962 | reviewed | CD63_HUMAN | CD63 antigen (Granulophysin) (Lysosomal-associated membrane protein 3) (LAMP-3) (Lysosome integral membrane protein 1) (Limp1) (Melanoma-associated antigen ME491) (OMA81H) (Ocular melanoma-associated antigen) (Tetraspanin-30) (Tspan-30) (CD antigen CD63) | CD63 MLA1 TSPAN30 | Homo sapiens (Human) | 238 |
| P0CG47 | reviewed | UBB_HUMAN | Polyubiquitin-B [Cleaved into: Ubiquitin] | UBB | Homo sapiens (Human) | 229 |
| P0CG48 | reviewed | UBC_HUMAN | Polyubiquitin-C [Cleaved into: Ubiquitin] | UBC | Homo sapiens (Human) | 685 |
| P0DPH7 | reviewed | TBA3C_HUMAN | Tubulin alpha-3C chain (EC 3.6.5.-) (Alpha-tubulin 2) (Alpha-tubulin 3C) (Tubulin alpha-2 chain) [Cleaved into: Detyrosinated tubulin alpha-3C chain] | TUBA3C TUBA2 | Homo sapiens (Human) | 450 |
| P0DPH8 | reviewed | TBA3D_HUMAN | Tubulin alpha-3D chain (EC 3.6.5.-) (Alpha-tubulin 3D) [Cleaved into: Detyrosinated tubulin alpha-3D chain] | TUBA3D | Homo sapiens (Human) | 450 |
| P40818 | reviewed | UBP8_HUMAN | Ubiquitin carboxyl-terminal hydrolase 8 (EC 3.4.19.12) (Deubiquitinating enzyme 8) (Ubiquitin isopeptidase Y) (hUBPy) (Ubiquitin thioesterase 8) (Ubiquitin-specific-processing protease 8) | USP8 KIAA0055 UBPY | Homo sapiens (Human) | 1118 |
| P40967 | reviewed | PMEL_HUMAN | Melanocyte protein PMEL (ME20-M) (ME20M) (Melanocyte protein Pmel 17) (Melanocytes lineage-specific antigen GP100) (Melanoma-associated ME20 antigen) (P1) (P100) (Premelanosome protein) (Silver locus protein homolog) [Cleaved into: M-alpha (95 kDa melanocyte-specific secreted glycoprotein) (P26) (Secreted melanoma-associated ME20 antigen) (ME20-S) (ME20S); M-beta] | PMEL D12S53E PMEL17 SILV | Homo sapiens (Human) | 661 |
| P46934 | reviewed | NEDD4_HUMAN | E3 ubiquitin-protein ligase NEDD4 (EC 2.3.2.26) (Cell proliferation-inducing gene 53 protein) (HECT-type E3 ubiquitin transferase NEDD4) (Neural precursor cell expressed developmentally down-regulated protein 4) (NEDD-4) | NEDD4 KIAA0093 NEDD4-1 RPF1 PIG53 | Homo sapiens (Human) | 1319 |
| P46940 | reviewed | IQGA1_HUMAN | Ras GTPase-activating-like protein IQGAP1 (p195) | IQGAP1 KIAA0051 | Homo sapiens (Human) | 1657 |
| P53990 | reviewed | IST1_HUMAN | IST1 homolog (hIST1) (Charged multivesicular body protein 8) (CHMP8) (Putative MAPK-activating protein PM28) | IST1 KIAA0174 | Homo sapiens (Human) | 364 |
| P57768 | reviewed | SNX16_HUMAN | Sorting nexin-16 | SNX16 | Homo sapiens (Human) | 344 |
| P61073 | reviewed | CXCR4_HUMAN | C-X-C chemokine receptor type 4 (CXC-R4) (CXCR-4) (FB22) (Fusin) (HM89) (LCR1) (Leukocyte-derived seven transmembrane domain receptor) (LESTR) (Lipopolysaccharide-associated protein 3) (LAP-3) (LPS-associated protein 3) (NPYRL) (Stromal cell-derived factor 1 receptor) (SDF-1 receptor) (CD antigen CD184) | CXCR4 | Homo sapiens (Human) | 352 |
| P62979 | reviewed | RS27A_HUMAN | Ubiquitin-ribosomal protein eS31 fusion protein (Ubiquitin carboxyl extension protein 80) [Cleaved into: Ubiquitin; Small ribosomal subunit protein eS31 (40S ribosomal protein S27a)] | RPS27A UBA80 UBCEP1 | Homo sapiens (Human) | 156 |
| P62987 | reviewed | RL40_HUMAN | Ubiquitin-ribosomal protein eL40 fusion protein (CEP52) (Ubiquitin A-52 residue ribosomal protein fusion product 1) [Cleaved into: Ubiquitin; Large ribosomal subunit protein eL40 (60S ribosomal protein L40)] | UBA52 UBCEP2 | Homo sapiens (Human) | 128 |
| P68363 | reviewed | TBA1B_HUMAN | Tubulin alpha-1B chain (EC 3.6.5.-) (Alpha-tubulin ubiquitous) (Tubulin K-alpha-1) (Tubulin alpha-ubiquitous chain) [Cleaved into: Detyrosinated tubulin alpha-1B chain] | TUBA1B | Homo sapiens (Human) | 451 |
| P68366 | reviewed | TBA4A_HUMAN | Tubulin alpha-4A chain (EC 3.6.5.-) (Alpha-tubulin 1) (Testis-specific alpha-tubulin) (Tubulin H2-alpha) (Tubulin alpha-1 chain) | TUBA4A TUBA1 | Homo sapiens (Human) | 448 |
| P68371 | reviewed | TBB4B_HUMAN | Tubulin beta-4B chain (Tubulin beta-2 chain) (Tubulin beta-2C chain) | TUBB4B TUBB2C | Homo sapiens (Human) | 445 |
| Q13464 | reviewed | ROCK1_HUMAN | Rho-associated protein kinase 1 (EC 2.7.11.1) (Renal carcinoma antigen NY-REN-35) (Rho-associated, coiled-coil-containing protein kinase 1) (Rho-associated, coiled-coil-containing protein kinase I) (ROCK-I) (p160 ROCK-1) (p160ROCK) | ROCK1 | Homo sapiens (Human) | 1354 |
| Q13509 | reviewed | TBB3_HUMAN | Tubulin beta-3 chain (Tubulin beta-4 chain) (Tubulin beta-III) | TUBB3 TUBB4 | Homo sapiens (Human) | 450 |
| Q13596 | reviewed | SNX1_HUMAN | Sorting nexin-1 | SNX1 | Homo sapiens (Human) | 522 |
| Q13885 | reviewed | TBB2A_HUMAN | Tubulin beta-2A chain (Tubulin beta class IIa) | TUBB2A TUBB2 | Homo sapiens (Human) | 445 |
| Q15036 | reviewed | SNX17_HUMAN | Sorting nexin-17 | SNX17 KIAA0064 | Homo sapiens (Human) | 470 |
| Q3ZCM7 | reviewed | TBB8_HUMAN | Tubulin beta-8 chain (Tubulin beta 8 class VIII) | TUBB8 | Homo sapiens (Human) | 444 |
| Q53EZ4 | reviewed | CEP55_HUMAN | Centrosomal protein of 55 kDa (Cep55) (Up-regulated in colon cancer 6) | CEP55 C10orf3 URCC6 | Homo sapiens (Human) | 464 |
| Q5T0F9 | reviewed | C2D1B_HUMAN | Coiled-coil and C2 domain-containing protein 1B (Five prime repressor element under dual repression-binding protein 2) (FRE under dual repression-binding protein 2) (Freud-2) | CC2D1B KIAA1836 | Homo sapiens (Human) | 858 |
| Q5T0T0 | reviewed | MARH8_HUMAN | E3 ubiquitin-protein ligase MARCHF8 (EC 2.3.2.27) (Cellular modulator of immune recognition) (c-MIR) (Membrane-associated RING finger protein 8) (Membrane-associated RING-CH protein VIII) (MARCH-VIII) (RING finger protein 178) (RING-type E3 ubiquitin transferase MARCHF8) | MARCHF8 MARCH8 MIR RNF178 | Homo sapiens (Human) | 291 |
| Q5VW32 | reviewed | BROX_HUMAN | BRO1 domain-containing protein BROX (BRO1 domain- and CAAX motif-containing protein) | BROX BROFTI C1orf58 | Homo sapiens (Human) | 411 |
| Q5VWJ9 | reviewed | SNX30_HUMAN | Sorting nexin-30 | SNX30 | Homo sapiens (Human) | 437 |
| Q6DKK2 | reviewed | TTC19_HUMAN | Tetratricopeptide repeat protein 19, mitochondrial (TPR repeat protein 19) | TTC19 | Homo sapiens (Human) | 380 |
| Q6PEY2 | reviewed | TBA3E_HUMAN | Tubulin alpha-3E chain (EC 3.6.5.-) (Alpha-tubulin 3E) [Cleaved into: Detyrosinated tubulin alpha-3E chain] | TUBA3E | Homo sapiens (Human) | 450 |
| Q6UWE0 | reviewed | LRSM1_HUMAN | E3 ubiquitin-protein ligase LRSAM1 (EC 2.3.2.27) (Leucine-rich repeat and sterile alpha motif-containing protein 1) (RING-type E3 ubiquitin transferase LRSAM1) (Tsg101-associated ligase) (hTAL) | LRSAM1 TAL UNQ6496/PRO21356 | Homo sapiens (Human) | 723 |
| Q71U36 | reviewed | TBA1A_HUMAN | Tubulin alpha-1A chain (EC 3.6.5.-) (Alpha-tubulin 3) (Tubulin B-alpha-1) (Tubulin alpha-3 chain) [Cleaved into: Detyrosinated tubulin alpha-1A chain] | TUBA1A TUBA3 | Homo sapiens (Human) | 451 |
| Q7LBR1 | reviewed | CHM1B_HUMAN | Charged multivesicular body protein 1b (CHMP1.5) (Chromatin-modifying protein 1b) (CHMP1b) (Vacuolar protein sorting-associated protein 46-2) (Vps46-2) (hVps46-2) | CHMP1B C18orf2 | Homo sapiens (Human) | 199 |
| Q7Z614 | reviewed | SNX20_HUMAN | Sorting nexin-20 (Selectin ligand-interactor cytoplasmic 1) (SLIC-1) | SNX20 SLIC1 | Homo sapiens (Human) | 316 |
| Q86VN1 | reviewed | VPS36_HUMAN | Vacuolar protein-sorting-associated protein 36 (ELL-associated protein of 45 kDa) (ESCRT-II complex subunit VPS36) | VPS36 C13orf9 EAP45 CGI-145 | Homo sapiens (Human) | 386 |
| Q86XE0 | reviewed | SNX32_HUMAN | Sorting nexin-32 (Sorting nexin-6B) | SNX32 SNX6B | Homo sapiens (Human) | 403 |
| Q86XT2 | reviewed | VP37D_HUMAN | Vacuolar protein sorting-associated protein 37D (ESCRT-I complex subunit VPS37D) (Williams-Beuren syndrome chromosomal region 24 protein) | VPS37D WBSCR24 | Homo sapiens (Human) | 251 |
| Q8IV08 | reviewed | PLD3_HUMAN | 5'-3' exonuclease PLD3 (EC 3.1.16.1) (Choline phosphatase 3) (HindIII K4L homolog) (Hu-K4) (Phosphatidylcholine-hydrolyzing phospholipase D3) (Phospholipase D3) (PLD 3) | PLD3 | Homo sapiens (Human) | 490 |
| Q8IX04 | reviewed | UEVLD_HUMAN | Ubiquitin-conjugating enzyme E2 variant 3 (UEV-3) (EV and lactate/malate dehydrogenase domain-containing protein) | UEVLD UEV3 | Homo sapiens (Human) | 471 |
| Q8N0X7 | reviewed | SPART_HUMAN | Spartin (Spastic paraplegia 20 protein) (Trans-activated by hepatitis C virus core protein 1) | SPART KIAA0610 SPG20 TAHCCP1 | Homo sapiens (Human) | 666 |
| Q8N5I2 | reviewed | ARRD1_HUMAN | Arrestin domain-containing protein 1 (Alpha-arrestin 1) | ARRDC1 | Homo sapiens (Human) | 433 |
| Q8NC56 | reviewed | LEMD2_HUMAN | LEM domain-containing protein 2 (hLEM2) | LEMD2 | Homo sapiens (Human) | 503 |
| Q8ND25 | reviewed | ZNRF1_HUMAN | E3 ubiquitin-protein ligase ZNRF1 (EC 2.3.2.27) (Nerve injury-induced gene 283 protein) (RING-type E3 ubiquitin transferase ZNRF1) (Zinc/RING finger protein 1) | ZNRF1 NIN283 | Homo sapiens (Human) | 227 |
| Q8NEZ2 | reviewed | VP37A_HUMAN | Vacuolar protein sorting-associated protein 37A (hVps37A) (ESCRT-I complex subunit VPS37A) (Hepatocellular carcinoma-related protein 1) | VPS37A HCRP1 | Homo sapiens (Human) | 397 |
| Q8TDB6 | reviewed | DTX3L_HUMAN | E3 ubiquitin-protein ligase DTX3L (EC 2.3.2.27) (B-lymphoma- and BAL-associated protein) (Protein deltex-3-like) (RING-type E3 ubiquitin transferase DTX3L) (Rhysin-2) (Rhysin2) | DTX3L BBAP | Homo sapiens (Human) | 740 |
| Q8WUM4 | reviewed | PDC6I_HUMAN | Programmed cell death 6-interacting protein (PDCD6-interacting protein) (ALG-2-interacting protein 1) (ALG-2-interacting protein X) (Hp95) | PDCD6IP AIP1 ALIX KIAA1375 | Homo sapiens (Human) | 868 |
| Q8WUX9 | reviewed | CHMP7_HUMAN | Charged multivesicular body protein 7 (Chromatin-modifying protein 7) | CHMP7 | Homo sapiens (Human) | 453 |
| Q8WV41 | reviewed | SNX33_HUMAN | Sorting nexin-33 (SH3 and PX domain-containing protein 3) | SNX33 SH3PX3 SH3PXD3C SNX30 | Homo sapiens (Human) | 574 |
| Q8WV92 | reviewed | MITD1_HUMAN | MIT domain-containing protein 1 | MITD1 | Homo sapiens (Human) | 249 |
| Q8WXH0 | reviewed | SYNE2_HUMAN | Nesprin-2 (KASH domain-containing protein 2) (KASH2) (Nuclear envelope spectrin repeat protein 2) (Nucleus and actin connecting element protein) (Protein NUANCE) (Synaptic nuclear envelope protein 2) (Syne-2) | SYNE2 KIAA1011 NUA | Homo sapiens (Human) | 6885 |
| Q92543 | reviewed | SNX19_HUMAN | Sorting nexin-19 | SNX19 KIAA0254 | Homo sapiens (Human) | 992 |
| Q92783 | reviewed | STAM1_HUMAN | Signal transducing adapter molecule 1 (STAM-1) | STAM STAM1 | Homo sapiens (Human) | 540 |
| Q96B67 | reviewed | ARRD3_HUMAN | Arrestin domain-containing protein 3 (TBP-2-like inducible membrane protein) (TLIMP) | ARRDC3 KIAA1376 | Homo sapiens (Human) | 414 |
| Q96CF2 | reviewed | CHM4C_HUMAN | Charged multivesicular body protein 4c (Chromatin-modifying protein 4c) (CHMP4c) (SNF7 homolog associated with Alix 3) (SNF7-3) (hSnf7-3) (Vacuolar protein sorting-associated protein 32-3) (Vps32-3) (hVps32-3) | CHMP4C SHAX3 | Homo sapiens (Human) | 233 |
| Q96EY5 | reviewed | MB12A_HUMAN | Multivesicular body subunit 12A (CIN85/CD2AP family-binding protein) (ESCRT-I complex subunit MVB12A) (Protein FAM125A) | MVB12A CFBP FAM125A | Homo sapiens (Human) | 273 |
| Q96FZ7 | reviewed | CHMP6_HUMAN | Charged multivesicular body protein 6 (Chromatin-modifying protein 6) (Vacuolar protein sorting-associated protein 20) (Vps20) (hVps20) | CHMP6 VPS20 | Homo sapiens (Human) | 201 |
| Q96GD4 | reviewed | AURKB_HUMAN | Aurora kinase B (EC 2.7.11.1) (Aurora 1) (Aurora- and IPL1-like midbody-associated protein 1) (AIM-1) (Aurora/IPL1-related kinase 2) (ARK-2) (Aurora-related kinase 2) (STK-1) (Serine/threonine-protein kinase 12) (Serine/threonine-protein kinase 5) (Serine/threonine-protein kinase aurora-B) | AURKB AIK2 AIM1 AIRK2 ARK2 STK1 STK12 STK5 | Homo sapiens (Human) | 344 |
| Q96H20 | reviewed | SNF8_HUMAN | Vacuolar-sorting protein SNF8 (ELL-associated protein of 30 kDa) (ESCRT-II complex subunit VPS22) (hVps22) | SNF8 EAP30 | Homo sapiens (Human) | 258 |
| Q96J02 | reviewed | ITCH_HUMAN | E3 ubiquitin-protein ligase Itchy homolog (Itch) (EC 2.3.2.26) (Atrophin-1-interacting protein 4) (AIP4) (HECT-type E3 ubiquitin transferase Itchy homolog) (NFE2-associated polypeptide 1) (NAPP1) | ITCH | Homo sapiens (Human) | 903 |
| Q96L92 | reviewed | SNX27_HUMAN | Sorting nexin-27 | SNX27 KIAA0488 My014 | Homo sapiens (Human) | 541 |
| Q96L93 | reviewed | KI16B_HUMAN | Kinesin-like protein KIF16B (Sorting nexin-23) | KIF16B C20orf23 KIAA1590 SNX23 | Homo sapiens (Human) | 1317 |
| Q96RF0 | reviewed | SNX18_HUMAN | Sorting nexin-18 (SH3 and PX domain-containing protein 3B) | SNX18 SH3PXD3B | Homo sapiens (Human) | 628 |
| Q99732 | reviewed | LITAF_HUMAN | Lipopolysaccharide-induced tumor necrosis factor-alpha factor (LPS-induced TNF-alpha factor) (Small integral membrane protein of lysosome/late endosome) (p53-induced gene 7 protein) | LITAF PIG7 SIMPLE | Homo sapiens (Human) | 161 |
| Q99816 | reviewed | TS101_HUMAN | Tumor susceptibility gene 101 protein (ESCRT-I complex subunit TSG101) | TSG101 | Homo sapiens (Human) | 390 |
| Q9BQE3 | reviewed | TBA1C_HUMAN | Tubulin alpha-1C chain (EC 3.6.5.-) (Alpha-tubulin 6) (Tubulin alpha-6 chain) [Cleaved into: Detyrosinated tubulin alpha-1C chain] | TUBA1C TUBA6 | Homo sapiens (Human) | 449 |
| Q9BRG1 | reviewed | VPS25_HUMAN | Vacuolar protein-sorting-associated protein 25 (hVps25) (Dermal papilla-derived protein 9) (ELL-associated protein of 20 kDa) (ESCRT-II complex subunit VPS25) | VPS25 DERP9 EAP20 | Homo sapiens (Human) | 176 |
| Q9BUF5 | reviewed | TBB6_HUMAN | Tubulin beta-6 chain (Tubulin beta class V) | TUBB6 | Homo sapiens (Human) | 446 |
| Q9BVA1 | reviewed | TBB2B_HUMAN | Tubulin beta-2B chain | TUBB2B | Homo sapiens (Human) | 445 |
| Q9BVK6 | reviewed | TMED9_HUMAN | Transmembrane emp24 domain-containing protein 9 (GMP25) (Glycoprotein 25L2) (p24 family protein alpha-2) (p24alpha2) (p25) | TMED9 GP25L2 | Homo sapiens (Human) | 235 |
| Q9BY43 | reviewed | CHM4A_HUMAN | Charged multivesicular body protein 4a (Chromatin-modifying protein 4a) (CHMP4a) (SNF7 homolog associated with Alix-2) (SNF7-1) (hSnf-1) (Vacuolar protein sorting-associated protein 32-1) (Vps32-1) (hVps32-1) | CHMP4A C14orf123 SHAX2 CDA04 HSPC134 | Homo sapiens (Human) | 222 |
| Q9H0M0 | reviewed | WWP1_HUMAN | NEDD4-like E3 ubiquitin-protein ligase WWP1 (EC 2.3.2.26) (Atrophin-1-interacting protein 5) (AIP5) (HECT-type E3 ubiquitin transferase WWP1) (TGIF-interacting ubiquitin ligase 1) (Tiul1) (WW domain-containing protein 1) | WWP1 | Homo sapiens (Human) | 922 |
| Q9H3S7 | reviewed | PTN23_HUMAN | Tyrosine-protein phosphatase non-receptor type 23 (EC 3.1.3.48) (His domain-containing protein tyrosine phosphatase) (HD-PTP) (Protein tyrosine phosphatase TD14) (PTP-TD14) | PTPN23 KIAA1471 | Homo sapiens (Human) | 1636 |
| Q9H444 | reviewed | CHM4B_HUMAN | Charged multivesicular body protein 4b (Chromatin-modifying protein 4b) (CHMP4b) (SNF7 homolog associated with Alix 1) (SNF7-2) (hSnf7-2) (Vacuolar protein sorting-associated protein 32-2) (Vps32-2) (hVps32-2) | CHMP4B C20orf178 SHAX1 | Homo sapiens (Human) | 224 |
| Q9H4B7 | reviewed | TBB1_HUMAN | Tubulin beta-1 chain | TUBB1 | Homo sapiens (Human) | 451 |
| Q9H7P6 | reviewed | MB12B_HUMAN | Multivesicular body subunit 12B (ESCRT-I complex subunit MVB12B) (Protein FAM125B) | MVB12B C9orf28 FAM125B | Homo sapiens (Human) | 319 |
| Q9H9H4 | reviewed | VP37B_HUMAN | Vacuolar protein sorting-associated protein 37B (hVps37B) (ESCRT-I complex subunit VPS37B) | VPS37B | Homo sapiens (Human) | 285 |
| Q9HD42 | reviewed | CHM1A_HUMAN | Charged multivesicular body protein 1a (Chromatin-modifying protein 1a) (CHMP1a) (Vacuolar protein sorting-associated protein 46-1) (Vps46-1) (hVps46-1) | CHMP1A CHMP1 KIAA0047 PCOLN3 PRSM1 | Homo sapiens (Human) | 196 |
| Q9NP79 | reviewed | VTA1_HUMAN | Vacuolar protein sorting-associated protein VTA1 homolog (Dopamine-responsive gene 1 protein) (DRG-1) (LYST-interacting protein 5) (LIP5) (SKD1-binding protein 1) (SBP1) | VTA1 C6orf55 HSPC228 My012 | Homo sapiens (Human) | 307 |
| Q9NRS6 | reviewed | SNX15_HUMAN | Sorting nexin-15 | SNX15 | Homo sapiens (Human) | 342 |
| Q9NY65 | reviewed | TBA8_HUMAN | Tubulin alpha-8 chain (EC 3.6.5.-) (Alpha-tubulin 8) (Tubulin alpha chain-like 2) [Cleaved into: Dephenylalaninated tubulin alpha-8 chain] | TUBA8 TUBAL2 | Homo sapiens (Human) | 449 |
| Q9NZ09 | reviewed | UBAP1_HUMAN | Ubiquitin-associated protein 1 (UBAP-1) (Nasopharyngeal carcinoma-associated gene 20 protein) | UBAP1 NAG20 | Homo sapiens (Human) | 502 |
| Q9NZZ3 | reviewed | CHMP5_HUMAN | Charged multivesicular body protein 5 (Chromatin-modifying protein 5) (SNF7 domain-containing protein 2) (Vacuolar protein sorting-associated protein 60) (Vps60) (hVps60) | CHMP5 C9orf83 SNF7DC2 CGI-34 HSPC177 PNAS-114 PNAS-2 | Homo sapiens (Human) | 219 |
| Q9UBP0 | reviewed | SPAST_HUMAN | Spastin (EC 5.6.1.1) (Spastic paraplegia 4 protein) | SPAST ADPSP FSP2 KIAA1083 SPG4 | Homo sapiens (Human) | 616 |
| Q9UHD2 | reviewed | TBK1_HUMAN | Serine/threonine-protein kinase TBK1 (EC 2.7.11.1) (NF-kappa-B-activating kinase) (T2K) (TANK-binding kinase 1) | TBK1 NAK | Homo sapiens (Human) | 729 |
| Q9UK41 | reviewed | VPS28_HUMAN | Vacuolar protein sorting-associated protein 28 homolog (H-Vps28) (ESCRT-I complex subunit VPS28) | VPS28 | Homo sapiens (Human) | 221 |
| Q9UMY4 | reviewed | SNX12_HUMAN | Sorting nexin-12 | SNX12 | Homo sapiens (Human) | 162 |
| Q9UN37 | reviewed | VPS4A_HUMAN | Vacuolar protein sorting-associated protein 4A (EC 3.6.4.6) (Protein SKD2) (VPS4-1) (hVPS4) | VPS4A VPS4 | Homo sapiens (Human) | 437 |
| Q9UNH6 | reviewed | SNX7_HUMAN | Sorting nexin-7 | SNX7 | Homo sapiens (Human) | 387 |
| Q9UNH7 | reviewed | SNX6_HUMAN | Sorting nexin-6 (TRAF4-associated factor 2) [Cleaved into: Sorting nexin-6, N-terminally processed] | SNX6 | Homo sapiens (Human) | 406 |
| Q9UQN3 | reviewed | CHM2B_HUMAN | Charged multivesicular body protein 2b (CHMP2.5) (Chromatin-modifying protein 2b) (CHMP2b) (Vacuolar protein sorting-associated protein 2-2) (Vps2-2) (hVps2-2) | CHMP2B CGI-84 | Homo sapiens (Human) | 213 |
| Q9Y250 | reviewed | LZTS1_HUMAN | Leucine zipper putative tumor suppressor 1 (F37/esophageal cancer-related gene-coding leucine-zipper motif) (Fez1) | LZTS1 FEZ1 | Homo sapiens (Human) | 596 |
| Q9Y263 | reviewed | PLAP_HUMAN | Phospholipase A-2-activating protein (PLA2P) (PLAP) | PLAA PLAP | Homo sapiens (Human) | 795 |
| Q9Y3E7 | reviewed | CHMP3_HUMAN | Charged multivesicular body protein 3 (Chromatin-modifying protein 3) (Neuroendocrine differentiation factor) (Vacuolar protein sorting-associated protein 24) (hVps24) | CHMP3 CGI149 NEDF VPS24 CGI-149 | Homo sapiens (Human) | 222 |
| Q9Y5K6 | reviewed | CD2AP_HUMAN | CD2-associated protein (Adapter protein CMS) (Cas ligand with multiple SH3 domains) | CD2AP | Homo sapiens (Human) | 639 |
| Q9Y5W7 | reviewed | SNX14_HUMAN | Sorting nexin-14 | SNX14 | Homo sapiens (Human) | 946 |
| Q9Y5W8 | reviewed | SNX13_HUMAN | Sorting nexin-13 (RGS domain- and PHOX domain-containing protein) (RGS-PX1) | SNX13 KIAA0713 | Homo sapiens (Human) | 968 |
| Q9Y5W9 | reviewed | SNX11_HUMAN | Sorting nexin-11 | SNX11 | Homo sapiens (Human) | 270 |
| Q9Y5X0 | reviewed | SNX10_HUMAN | Sorting nexin-10 | SNX10 | Homo sapiens (Human) | 201 |
| Q9Y5X2 | reviewed | SNX8_HUMAN | Sorting nexin-8 | SNX8 | Homo sapiens (Human) | 465 |
| Q9Y5X3 | reviewed | SNX5_HUMAN | Sorting nexin-5 | SNX5 | Homo sapiens (Human) | 404 |
| B2RE76 | unreviewed | B2RE76_HUMAN | Chromatin modifying protein 2B (cDNA FLJ11303 fis, clone PLACE1009995, highly similar to Charged multivesicular body protein 2b) (cDNA FLJ11318 fis, clone PLACE1010274, highly similar to Charged multivesicular body protein 2b) (cDNA, FLJ95104) | CHMP2B hCG_27849 | Homo sapiens (Human) | 213 |
| P59074 | reviewed | CHM4P_HUMAN | Putative charged multivesicular body protein 4B-like protein CHMP4BP1 (Charged multivesicular body protein 4B pseudogene 1) | CHMP4BP1 CGI-301 | Homo sapiens (Human) | 171 |
| Q548N1 | unreviewed | Q548N1_HUMAN | Vacuolar protein sorting-associated protein 28 homolog | VPS28 hCG_24005 | Homo sapiens (Human) | 221 |
| Q8TBH0 | reviewed | ARRD2_HUMAN | Arrestin domain-containing protein 2 | ARRDC2 PP2703 | Homo sapiens (Human) | 407 |
| Q969T3 | reviewed | SNX21_HUMAN | Sorting nexin-21 (Sorting nexin L) (SNX-L) | SNX21 C20orf161 SNXL | Homo sapiens (Human) | 373 |
| Q96L94 | reviewed | SNX22_HUMAN | Sorting nexin-22 | SNX22 | Homo sapiens (Human) | 193 |
| Q9H3E2 | reviewed | SNX25_HUMAN | Sorting nexin-25 | SNX25 MSTP043 | Homo sapiens (Human) | 840 |
| A0A087WW88 | unreviewed | A0A087WW88_HUMAN | Charged multivesicular body protein 2B | CHMP2B | Homo sapiens (Human) | 229 |
| A0A140T963 | unreviewed | A0A140T963_HUMAN | RNF103-CHMP3 readthrough | RNF103-CHMP3 | Homo sapiens (Human) | 221 |
| A0A6Q8PF35 | unreviewed | A0A6Q8PF35_HUMAN | Charged multivesicular body protein 1A | CHMP1A | Homo sapiens (Human) | 154 |
| A0A6Q8PFF9 | unreviewed | A0A6Q8PFF9_HUMAN | Charged multivesicular body protein 1A | CHMP1A | Homo sapiens (Human) | 383 |
| A0A6Q8PFX8 | unreviewed | A0A6Q8PFX8_HUMAN | Charged multivesicular body protein 1A | CHMP1A | Homo sapiens (Human) | 132 |
| A0A6Q8PG85 | unreviewed | A0A6Q8PG85_HUMAN | Charged multivesicular body protein 1A | CHMP1A | Homo sapiens (Human) | 199 |
| A0A6Q8PGM8 | unreviewed | A0A6Q8PGM8_HUMAN | Charged multivesicular body protein 1A | CHMP1A | Homo sapiens (Human) | 113 |
| A0A6Q8PHU0 | unreviewed | A0A6Q8PHU0_HUMAN | Charged multivesicular body protein 1A | CHMP1A | Homo sapiens (Human) | 147 |
| A0A7I2V2D8 | unreviewed | A0A7I2V2D8_HUMAN | Hepatocyte growth factor-regulated tyrosine kinase substrate | HGS | Homo sapiens (Human) | 767 |
| A0A7I2V2K2 | unreviewed | A0A7I2V2K2_HUMAN | Hepatocyte growth factor-regulated tyrosine kinase substrate | HGS | Homo sapiens (Human) | 737 |
| A0A7I2V2R9 | unreviewed | A0A7I2V2R9_HUMAN | Hepatocyte growth factor-regulated tyrosine kinase substrate | HGS | Homo sapiens (Human) | 768 |
| A0A7I2V2W5 | unreviewed | A0A7I2V2W5_HUMAN | Hepatocyte growth factor-regulated tyrosine kinase substrate | HGS | Homo sapiens (Human) | 768 |
| A0A7I2V307 | unreviewed | A0A7I2V307_HUMAN | Hepatocyte growth factor-regulated tyrosine kinase substrate | HGS | Homo sapiens (Human) | 783 |
| A0A7I2V3F9 | unreviewed | A0A7I2V3F9_HUMAN | Hepatocyte growth factor-regulated tyrosine kinase substrate | HGS | Homo sapiens (Human) | 765 |
| A0A7I2V3I8 | unreviewed | A0A7I2V3I8_HUMAN | Hepatocyte growth factor-regulated tyrosine kinase substrate | HGS | Homo sapiens (Human) | 761 |
| A0A7I2V3J3 | unreviewed | A0A7I2V3J3_HUMAN | Hepatocyte growth factor-regulated tyrosine kinase substrate | HGS | Homo sapiens (Human) | 777 |
| A0A7I2V3Z1 | unreviewed | A0A7I2V3Z1_HUMAN | Hepatocyte growth factor-regulated tyrosine kinase substrate | HGS | Homo sapiens (Human) | 690 |
| A0A7I2V5A3 | unreviewed | A0A7I2V5A3_HUMAN | Hepatocyte growth factor-regulated tyrosine kinase substrate | HGS | Homo sapiens (Human) | 800 |
| A0A7I2V637 | unreviewed | A0A7I2V637_HUMAN | Hepatocyte growth factor-regulated tyrosine kinase substrate | HGS | Homo sapiens (Human) | 795 |
| A0A7I2V6E0 | unreviewed | A0A7I2V6E0_HUMAN | Hepatocyte growth factor-regulated tyrosine kinase substrate | HGS hCG_1987560 | Homo sapiens (Human) | 710 |
| B2RA72 | unreviewed | B2RA72_HUMAN | cDNA, FLJ94734, Homo sapiens CHMP1.5 protein (CHMP1.5), mRNA |  | Homo sapiens (Human) | 196 |
| B3KMN6 | unreviewed | B3KMN6_HUMAN | cDNA FLJ11714 fis, clone HEMBA1005219, highly similar to Protein CHMP7 |  | Homo sapiens (Human) | 209 |
| B3KRZ9 | unreviewed | B3KRZ9_HUMAN | cDNA FLJ35181 fis, clone PLACE6015051, highly similar to Protein CHMP7 |  | Homo sapiens (Human) | 343 |
| B3KUH0 | unreviewed | B3KUH0_HUMAN | cDNA FLJ39883 fis, clone SPLEN2016268, highly similar to Protein CHMP7 |  | Homo sapiens (Human) | 453 |
| B4DXN3 | unreviewed | B4DXN3_HUMAN | cDNA FLJ57657, highly similar to Charged multivesicular body protein 1a |  | Homo sapiens (Human) | 137 |
| B4E1E2 | unreviewed | B4E1E2_HUMAN | Hepatocyte growth factor-regulated tyrosine kinase substrate |  | Homo sapiens (Human) | 661 |
| B7Z6C9 | unreviewed | B7Z6C9_HUMAN | Transmembrane 9 superfamily member |  | Homo sapiens (Human) | 815 |
| C9J0A7 | unreviewed | C9J0A7_HUMAN | Charged multivesicular body protein 2B | CHMP2B | Homo sapiens (Human) | 183 |
| E9PQ80 | unreviewed | E9PQ80_HUMAN | Charged multivesicular body protein 4A | CHMP4A | Homo sapiens (Human) | 213 |
| E9PQI5 | unreviewed | E9PQI5_HUMAN | Charged multivesicular body protein 4A | CHMP4A | Homo sapiens (Human) | 123 |
| E9PSI1 | unreviewed | E9PSI1_HUMAN | Transmembrane 9 superfamily member |  | Homo sapiens (Human) | 815 |
| F5H442 | unreviewed | F5H442_HUMAN | Tumor susceptibility 101 | TSG101 | Homo sapiens (Human) | 365 |
| F5H875 | unreviewed | F5H875_HUMAN | Charged multivesicular body protein 1A | CHMP1A | Homo sapiens (Human) | 253 |
| F8VUA2 | unreviewed | F8VUA2_HUMAN | Charged multivesicular body protein 1A | CHMP1A | Homo sapiens (Human) | 181 |
| F8VVT7 | unreviewed | F8VVT7_HUMAN | Charged multivesicular body protein 1A | CHMP1A | Homo sapiens (Human) | 187 |
| H0YBN0 | unreviewed | H0YBN0_HUMAN | VPS37A subunit of ESCRT-I | VPS37A | Homo sapiens (Human) | 170 |
| H0YIN6 | unreviewed | H0YIN6_HUMAN | Charged multivesicular body protein 4A | CHMP4A | Homo sapiens (Human) | 117 |
| I3L3E4 | unreviewed | I3L3E4_HUMAN | Charged multivesicular body protein 6 | CHMP6 | Homo sapiens (Human) | 113 |
| I3L4A1 | unreviewed | I3L4A1_HUMAN | Charged multivesicular body protein 6 | CHMP6 | Homo sapiens (Human) | 224 |
| I3L4G8 | unreviewed | I3L4G8_HUMAN | Charged multivesicular body protein 6 | CHMP6 | Homo sapiens (Human) | 173 |
| M0QXX8 | unreviewed | M0QXX8_HUMAN | Charged multivesicular body protein 2a | CHMP2A | Homo sapiens (Human) | 55 |
| M0R1L7 | unreviewed | M0R1L7_HUMAN | Charged multivesicular body protein 2a | CHMP2A | Homo sapiens (Human) | 159 |
| M0R1T5 | unreviewed | M0R1T5_HUMAN | Charged multivesicular body protein 2A | CHMP2A | Homo sapiens (Human) | 265 |
| Q8TEQ0 | reviewed | SNX29_HUMAN | Sorting nexin-29 (RUN domain-containing protein 2A) | SNX29 RUNDC2A | Homo sapiens (Human) | 813 |
| Q9H853 | reviewed | TBA4B_HUMAN | Putative tubulin-like protein alpha-4B (EC 3.6.5.-) (Alpha-tubulin 4B) | TUBA4B TUBA4 | Homo sapiens (Human) | 241 |
| Q9Y343 | reviewed | SNX24_HUMAN | Sorting nexin-24 | SNX24 SBBI31 UNQ654/PRO1284 | Homo sapiens (Human) | 169 |
| A0A0S2Z5H5 | unreviewed | A0A0S2Z5H5_HUMAN | Vacuolar protein sorting-associated protein 28 homolog | VPS28 | Homo sapiens (Human) | 204 |
| A0A3B3ISW7 | unreviewed | A0A3B3ISW7_HUMAN | Vacuolar protein-sorting-associated protein 36 (ESCRT-II complex subunit VPS36) | VPS36 | Homo sapiens (Human) | 324 |
| A0A6G6AA68 | unreviewed | A0A6G6AA68_HUMAN | Ubiquitin associated protein 1 | UBAP1 | Homo sapiens (Human) | 502 |
| A0A7I2V5A2 | unreviewed | A0A7I2V5A2_HUMAN | Hepatocyte growth factor-regulated tyrosine kinase substrate | HGS | Homo sapiens (Human) | 757 |
| A6NMU3 | unreviewed | A6NMU3_HUMAN | Signal transducing adaptor molecule | STAM | Homo sapiens (Human) | 173 |
| A8K0C2 | unreviewed | A8K0C2_HUMAN | Multivesicular body subunit 12A (ESCRT-I complex subunit MVB12A) (Protein FAM125A) |  | Homo sapiens (Human) | 273 |
| B2RAY1 | unreviewed | B2RAY1_HUMAN | cDNA, FLJ95184, highly similar to Homo sapiens signal transducing adaptor molecule (SH3 domain and ITAM motif) 1 (STAM), mRNA |  | Homo sapiens (Human) | 540 |
| B4DYD9 | unreviewed | B4DYD9_HUMAN | cDNA FLJ61465, highly similar to Homo sapiens vacuolar protein sorting 37C (VPS37C), mRNA |  | Homo sapiens (Human) | 322 |
| B4DZT2 | unreviewed | B4DZT2_HUMAN | cDNA FLJ57683, highly similar to Signal transducing adapter molecule 1 |  | Homo sapiens (Human) | 429 |
| C9JYT9 | unreviewed | C9JYT9_HUMAN | VPS37D subunit of ESCRT-I | VPS37D | Homo sapiens (Human) | 166 |
| E9PM90 | unreviewed | E9PM90_HUMAN | Vacuolar protein sorting-associated protein 28 homolog | VPS28 | Homo sapiens (Human) | 185 |
| E9PQR7 | unreviewed | E9PQR7_HUMAN | Vacuolar protein sorting-associated protein 28 homolog | VPS28 | Homo sapiens (Human) | 161 |
| E9PR04 | unreviewed | E9PR04_HUMAN | Vacuolar protein sorting-associated protein 28 homolog | VPS28 | Homo sapiens (Human) | 53 |
| F5GYI3 | reviewed | UBA1L_HUMAN | Ubiquitin-associated protein 1-like (UBAP-1L) | UBAP1L | Homo sapiens (Human) | 381 |
| F5H1F6 | unreviewed | F5H1F6_HUMAN | VPS37B subunit of ESCRT-I | VPS37B | Homo sapiens (Human) | 184 |
| F5H4M0 | unreviewed | F5H4M0_HUMAN | VPS37B subunit of ESCRT-I | VPS37B | Homo sapiens (Human) | 271 |
| F5H4Q5 | unreviewed | F5H4Q5_HUMAN | VPS37C subunit of ESCRT-I | VPS37C | Homo sapiens (Human) | 148 |
| K7ENE3 | unreviewed | K7ENE3_HUMAN | Vacuolar protein-sorting-associated protein 25 (ESCRT-II complex subunit VPS25) | VPS25 | Homo sapiens (Human) | 108 |
| Q59H03 | unreviewed | Q59H03_HUMAN | Vacuolar protein-sorting-associated protein 36 (ESCRT-II complex subunit VPS36) |  | Homo sapiens (Human) | 224 |
| A0A0D9SG79 | unreviewed | A0A0D9SG79_HUMAN | Ubiquitin associated protein 1 | UBAP1 | Homo sapiens (Human) | 528 |
| B3KW95 | unreviewed | B3KW95_HUMAN | cDNA FLJ42616 fis, clone BRACE3014317, highly similar to Homo sapiens vacuolar protein sorting 37A (yeast) (VPS37A), mRNA |  | Homo sapiens (Human) | 168 |
| B7Z1P9 | unreviewed | B7Z1P9_HUMAN | cDNA FLJ53722 |  | Homo sapiens (Human) | 304 |
| B7Z4X0 | unreviewed | B7Z4X0_HUMAN | cDNA FLJ55114 |  | Homo sapiens (Human) | 214 |
| D6RBI1 | unreviewed | D6RBI1_HUMAN | SNF8 subunit of ESCRT-II | SNF8 | Homo sapiens (Human) | 103 |
| D6RFY6 | unreviewed | D6RFY6_HUMAN | Vacuolar-sorting protein SNF8 (ESCRT-II complex subunit VPS22) | SNF8 | Homo sapiens (Human) | 167 |
| D6RJ86 | unreviewed | D6RJ86_HUMAN | Vacuolar-sorting protein SNF8 (ESCRT-II complex subunit VPS22) | SNF8 | Homo sapiens (Human) | 141 |
| E5RG91 | unreviewed | E5RG91_HUMAN | VPS37A subunit of ESCRT-I | VPS37A | Homo sapiens (Human) | 317 |
| E5RHB8 | unreviewed | E5RHB8_HUMAN | VPS37A subunit of ESCRT-I | VPS37A | Homo sapiens (Human) | 76 |
| E5RJ10 | unreviewed | E5RJ10_HUMAN | VPS37A subunit of ESCRT-I | VPS37A | Homo sapiens (Human) | 53 |
| E5RJX6 | unreviewed | E5RJX6_HUMAN | VPS37A subunit of ESCRT-I | VPS37A | Homo sapiens (Human) | 116 |
| E9PF00 | unreviewed | E9PF00_HUMAN | IST1 factor associated with ESCRT-III | IST1 | Homo sapiens (Human) | 41 |
| E9PJW8 | unreviewed | E9PJW8_HUMAN | Multivesicular body subunit 12A | MVB12A | Homo sapiens (Human) | 199 |
| E9PLL0 | unreviewed | E9PLL0_HUMAN | Multivesicular body subunit 12A | MVB12A | Homo sapiens (Human) | 121 |
| E9PLM9 | unreviewed | E9PLM9_HUMAN | VPS28 subunit of ESCRT-I | VPS28 | Homo sapiens (Human) | 35 |
| E9PLZ8 | unreviewed | E9PLZ8_HUMAN | Multivesicular body subunit 12A (ESCRT-I complex subunit MVB12A) (Protein FAM125A) | MVB12A | Homo sapiens (Human) | 133 |
| E9PQA6 | unreviewed | E9PQA6_HUMAN | Multivesicular body subunit 12A | MVB12A | Homo sapiens (Human) | 273 |
| F8W922 | unreviewed | F8W922_HUMAN | Multivesicular body subunit 12B | MVB12B | Homo sapiens (Human) | 205 |
| H0Y8S5 | unreviewed | H0Y8S5_HUMAN | SNF8 subunit of ESCRT-II | SNF8 | Homo sapiens (Human) | 81 |
| H0YKG2 | unreviewed | H0YKG2_HUMAN | Ubiquitin associated protein 1 like | UBAP1L | Homo sapiens (Human) | 117 |
| H3BQ38 | unreviewed | H3BQ38_HUMAN | IST1 factor associated with ESCRT-III | IST1 | Homo sapiens (Human) | 40 |
| I3L2X5 | unreviewed | I3L2X5_HUMAN | SNF8 subunit of ESCRT-II | SNF8 | Homo sapiens (Human) | 53 |
| I3L457 | unreviewed | I3L457_HUMAN | SNF8 subunit of ESCRT-II | SNF8 | Homo sapiens (Human) | 36 |
| J3QKS4 | unreviewed | J3QKS4_HUMAN | Tumor susceptibility 101 | TSG101 | Homo sapiens (Human) | 77 |
| J3QQP8 | unreviewed | J3QQP8_HUMAN | IST1 factor associated with ESCRT-III | IST1 | Homo sapiens (Human) | 85 |
| J3QRU6 | unreviewed | J3QRU6_HUMAN | Tumor susceptibility 101 | TSG101 | Homo sapiens (Human) | 63 |
| K7EKV4 | unreviewed | K7EKV4_HUMAN | Vacuolar protein-sorting-associated protein 25 (ESCRT-II complex subunit VPS25) | VPS25 | Homo sapiens (Human) | 111 |
| K7EP45 | unreviewed | K7EP45_HUMAN | Vacuolar protein-sorting-associated protein 25 (ESCRT-II complex subunit VPS25) | VPS25 | Homo sapiens (Human) | 99 |
| K7EPV2 | unreviewed | K7EPV2_HUMAN | SNF8 subunit of ESCRT-II | SNF8 | Homo sapiens (Human) | 16 |
| M0QZK6 | unreviewed | M0QZK6_HUMAN | Multivesicular body subunit 12A (ESCRT-I complex subunit MVB12A) (Protein FAM125A) | MVB12A | Homo sapiens (Human) | 122 |
| Q71MH5 | unreviewed | Q71MH5_HUMAN | Uncharacterized protein FP3517 | FP3517 | Homo sapiens (Human) | 133 |
| Q9H7N7 | unreviewed | Q9H7N7_HUMAN | FLJ00031 protein | FLJ00031 | Homo sapiens (Human) | 188 |
