# Supplementary material for: Neoliensinine promotes lysosomal cell death in T cell malignancies via a novel STING-TUBA1B pathway
Source: Genes Dis. 2025 Aug 14;13(6):101809. doi: 10.1016/j.gendis.2025.101809 (PMC13485499; doi:10.1016/j.gendis.2025.101809)
Supplement: Supplementary file 3 [file mmc3.docx]

**Supplementary Methods and Materials**

**Cell viability**

MTT powder was dissolved in PBS in 5 mg/mL and filtered using a 0.22 μm filter. 20 μL of MTT solution was added to each well of 96-well plates containing cells after reagent treatment and incubated for 4 h at 37 °C. Cells treated with equivalent amounts of dimethyl sulfoxide (DMSO; D2650, Sigma-Aldrich) were served as negative controls. Absorbance was read at 570 nm using a Synergy™ HT multi-mode reader (Bio-Tek, Winooski, VT, USA). The average optical density (OD) of five wells was used to determine cell viability according to the formula: $Inhibition rates (\%)=(1-\frac{\mathrm{OD}_{treatment group}}{\mathrm{OD}_{control group}})\times100\%$. *IC*_50_ values, representing the concentration causing 50% inhibition of cell viability, were calculated using the *logit* method through GraphPad Prism 8.0 (Boston, MA, USA).

**Cell death rates**

Cells were collected and labeled with Annexin V and PI according to the protocols of the Annexin V/PI Cell Apoptosis Detection Kit (A211-02; Vazyme Biotec, Nanjing, China) ^[1]^. Fluorescence was detected using a Becton Dickinson FACS Calibur flow cytometer (NJ, USA) and data analysis was performed using FlowJo software (Tree Star, Ashland, OR, USA).

**Western blot**

Whole cell lysates were extracted from cells after treatment with RIPA buffer (89901, ThermoFisher). Protein concentration was determined using the BCA Protein Assay Kit (23227, ThermoFisher). Equal amounts of protein lysate were resolved on SDS-PAGE gels and transferred to nitrocellulose membranes for Western blotting. Immunodetection was performed using an enhanced chemiluminescence (ECL) system (36222ES60, YEASEN). Chemiluminescence was detected using an Amersham Imager 600 (General Electric Company, Schenectady, NY, USA). The blots represent multiple independent experiments. Protein quantification was performed using Adobe Photoshop software (San Jose, CA, USA). Protein expression was quantified and normalized as follows: $Target protein expression \left( \% \right)=\frac{\left( \mathrm{GS}_{\mathrm{tb}}－\mathrm{GS}_{\mathrm{btb}} \right)\times A_{\mathrm{tb}}}{\left( \mathrm{GS}_{\mathrm{lcb}}－\mathrm{GS}_{\mathrm{blc}} \right)\times A_{\mathrm{lcb}}}\times100\%$. Where: GS = Gray Scale; A = Area; tb = target band; btb = background of target band; lcb = loading control band; blc = background of loading control.

**Cellular thermal shift assay (CETSA)**

The binding affinity of NeoL and STING was detected by CETSA. Approximately 2 × 10^6^ cells were incubated with 4 μmol/L of NeoL or an equal amount of DMSO for 30 min. Cells were then collected in PBS, divided into seven parts, and each pair was treated at a different temperature (37 °C, 45 °C, 47 °C, 49 °C, 51 °C, 52 °C, and 53 °C) for 3 min. Total protein was extracted by freezing and thawing three times in liquid nitrogen. Proteins were proceeded with Western blot protocols. The ligand-bound proteins are expected to be more resistant to high temperatures than unbound proteins, resulting in higher contents in the protein extracts.

**Drug affinity responsive target stability (DARTS)**

Approximately 1 × 10^7^ cells were lysed with M-PER buffer (ThermoFisher Scientific, Waltham, MA, USA) at 4 °C for 10 min. After centrifugation, 1 × TNC buffer (50 mmol/L Tris, 140 mmol/L NaCl, 10 mmol/L CaCl_2_) was added to the cell lysate. The lysate was divided equally into different groups, and 40 μmol/L of NeoL or 80 μmol/L of IsoL were added. After overnight rotation at 4 °C, the different groups of lysates were digested with pronase (Roche, Basel, Switzerland) at 1:4800 or 1:2400 dilution at 37 °C for 30 min. Proteolysis was quenched by mixing with 4 × loading buffer and boiled for 10 min. Differential proteins were then detected by western blot.

**Molecular docking**

The structural model of STING (PDB ID, 6NT6) was used to determine the interactions with NeoL, and the results were analyzed using AutoDock Vina software (the Scripps Research Institute, USA).

**Real time quantitative PCR (RT-qPCR) analysis**

Total RNA was extracted using FreeZol Reagent (R711-01, Vazyme biotec). One microgram of total RNA was used to transcribe the first strand cDNA with HiScript IV RT SuperMix for qPCR (R423-01, Vazyme biotec). RT-qPCR was performed using SYBR Green PCR Master Mix (Q131-02, Vazyme biotec) and specific PCR primers (GENEWIZ, Suzhou, China):

*STING*:

F: 5’-CCTGAGTCTCAGAACAACTGCC-3’;

R: 5’-GGTCTTCAAGCTGCCCACAGTA-3’.

*LC3*:

F: 5’-GAACGATACAAGGGTGAGAAGC-3’;

R: 5’-TACACCTCTGAGATTGGTGTGG-3’.

*P62*:

F: 5’-GAGAGTGTGGCAGCTGCCCT-3’;

R: 5’-GGCAGCTTCCTTCAGCCCTG-3’.

*GAPDH:*

F: 5’-AGCCACATCGCTCAGACAC-3’;

R: 5’-GCCCAATACGACCAAATCC-3’.

*TUBA1B:*

5’-CCTTCGCCTCCTAATCCCTA-3’;

5’-CCGTGTTCCAGGCAGTAGA-3’.

**Immunofluorescence**

Cells were collected and washed by PBS for 2 times and smeared on the cover glass. They were fixed in ice-cold methanol for 15 min and permeabilized in 0.17% Triton X-100 (X100-500 ML, Sigma Aldrich) for 17 min. After blocking with 3% bovine serum albumin (A3858-100G, Sigma Aldrich) for 1 h at room temperature, the cells were incubated with primary antibody overnight at 4 °C, followed by incubated with Alexa Fluor secondary antibody for 1 h and DAPI. The cells were imaged with a confocal laser scanning microscope (Fluoview FV1000, Olympus, Tokyo, Japan).

For cholesterol detection, cells treated with NeoL were fixed with 3% paraformaldehyde for 20 min, washed with PBS, and incubated with 1.5 mg/mL glycine for 10 min. Cells were then incubated with 50 mg/mL Filipin III (B6034, APExBIO Technology, TX, USA) containing 10% FBS for 2 h at room temperature in the dark, followed by confocal imaging.

Colocalization of different channel of the color was analyzed by ImageJ software (National Institutes of Health, USA). After drawing line by “straight” and analyzed by “Plugins-Graphics-RGB profile plot”, the signal intensity of RGB channel on the line was visualized. By statistical analysis of the area of a specific fluorescence color, the fluorescence was quantitatively analyzed. Using “Selection-Color range” in Photoshop software, the specific fluorescence color could be selected, and then by using “Measurement record”, the area of fluorescence was record.

**Cell transfection**

For RNA interference using lentiviral vectors, *TUBA1B* shRNA constructs and a negative control construct in the pLKO.1 vector system were purchased from Corues Biotechnology (Nanjing, China). For STING knockout (KO) using CRISPR-Cas9, pLV1cmv-Cas9-Puro-U6-sgRNA constructs were obtained from Corues Biotechnology. 293T cells were plated in 12-well dishes and co-transfected with shRNA constructs (10 μg) along with Lentiviral Mix (10 μL) and HG Transgene™ Reagent (60 μL) following the manufacturer’s instructions for the Lentiviral Packaging Kit (YEASEN 41102ES20) for 48 h. Viral stocks were harvested, filtered to remove non-adherent 293T cells, and used for transfection. For LC3-GFP-mCherry transfection, the commercial virus was purchased from HanBio (Shanghai, China). Jurkat cells transfected with Galectin-3-GFP (Gal3-GFP) stably were provided by Dr. Yingjie Qing from Nanjing University of Chinese Medicine ^[2]^. To select Jurkat cells that were stably transfected, cells were incubated in RPMI-1640 medium with 10% FBS and 2 mg/mL of puromycin for 48 h after lentivirus infection. After cells selection, pooled clones with stable transfection were harvested for use. For knockdown approach, the shRNA sequence included:

*TUBA1B* shRNA #1:

F: gatccGCTGTGTTTGTAGACTTGGAActcgagTTCCAAGTCTACAAACACAGCtttttt;

R: aattaaaaaaGCTGTGTTTGTAGACTTGGAActcgagTTCCAAGTCTACAAACACAGCg.

*TUBA1B* shRNA #2:

F: gatccGCTGAGAAAGCCTACCATGAActcgagTTCATGGTAGGCTTTCTCAGCtttttt;

R: aattaaaaaaGCTGAGAAAGCCTACCATGAActcgagTTCATGGTAGGCTTTCTCAGCg.

*TUBA1B* shRNA #3:

F: gatccgCCACAAGTTTGACCTGATGTActcgagTACATCAGGTCAAACTTGTGGtttttt;

R: aattaaaaaaCCACAAGTTTGACCTGATGTActcgagTACATCAGGTCAAACTTGTGGcg.

For Crispr-Cas9 knockout, the following gRNA were used:

STING KO#1:

CATATTACATCGGATATCTG.

STING KO#2:

GCATTACAACAACCTGCTAC.

STING KO#3:

GCATGGTCACCGGTCTGCTG.

**Lysosomal damage**

Galectin-3 puncta formation was used as a marker of lysosomal damage. Cells with stable expression of Galectin-3-GFP showed bright green fluorescence puncta when lysosomal damage occurred. Galectin-3 puncta positive cells were counted and the proportion of Galectin-3 puncta positive cells quantified. The number of Galectin-3 puncta in each cell, the quantity and area of Galectin-3 puncta were calculated with the aid of Photoshop software. Both sets of data were used to evaluate the degree of lysosomal damage in cells.

**Lysosomal enlargement**

Cells were stained with 0.5 mmol/L LysoTracker RED for 45 min in cell culture medium at 37 °C, and washed with PBS, suspended PBS and analyzed by flow cytometry or detected by confocal laser scanning microscope to analyze the lysosomal mass.

**Calcium level detection**

Cytoplasmic calcium levels were determined by fluorescence probe Fluo3-AM. After treatment, cells were incubated with 1 mmol/L of Fluo-3-AM in PBS at 37 °C for 40 min, then washed and resuspended in PBS. Cells were analyzed by flow cytometry at 488 nm.

***In vivo* examination**

The project was approved by the Ethical Committee of Nanjing University of Chinese Medicine (202412A009). For *in vivo* experiments, intraperitoneal administration formulation of NeoL (1 mg/mL in normal saline, 0.22 μm filtered) was prepared. Female *C57BL/6* mice (5 - 6 weeks, 18-20 g, Qinglong Mountain Animal Breeding Farm, Nanjing, China) were grafted with tumor cells suspended in serum-free DMEM medium. Cells (1 × 10^6^) were inoculated into the right legs of mice, which were then divided by body weight and received treatment, including Control group (n=6) injected with saline, NeoL group (n=6, *i.p.*) treated with 5 mg/kg NeoL, Dex group (n=6, *i.p.*) treated with 7.5 mg/kg dexamethasone - a positive agent, and Blank group (n=3) of normal mice with no injection of cells or drug. Mice treated with Dex or NeoL once for every day, starting from the day of EL4-grafting. For control group, mice received an equal volume of saline. Mice in blank group received normal feeding. The tumor grew hemispherically under the skin, and tumor volumes were measured every day. Tumor diameter was measured and tumor volume (mm^3^) was calculated as follows: $V_{\mathrm{Tumor}}=\frac{{(Shortest diameter)}^{2}\times(Longest diameter)}{2}$. At the end of the experimental period, mice were humanely euthanized, the tumors were dissected, and main organs were histologically processed. The tissue sections of main organs were stained with hematoxylin-eosin (H&E) and histopathological analysis were performed (Haixingli Biotechnology Co., Ltd, Nanjing, China).

**Co-immunoprecipitation (Co-IP) and CoIP-liquid chromatography mass spectrometry (LC-MS/MS)**

The lysates of cells (2 × 10^5^ counts) were extracted by RIPA buffer, and then incubated vertically with 1 μL of antibody overnight. Protein A/G Magnetic Beads (40 μL, MedChemExpress) were added and incubated for 4 h. The protein-antibody-Magnetic Beads complex was washed by RIPA buffer for three times, boiled with loading buffer, and separated with SDS-PAGE. A portion of the protein was analyzed by Western blot. For LC-MS/MS analysis, proteins were detected by sliver staining (Protein Stains K, C500021-0010, Sangon Biotech, Shanghai, China) and LC-MS/MS analysis (Lumingbio, Shanghai, China).

References

[1] Hu P, Li H, Sun W, et al. Cholesterol-associated lysosomal disorder triggers cell death of hematological malignancy: Dynamic analysis on cytotoxic effects of LW-218[J]. Acta Pharm Sin B, 2021, 11(10): 3178-3192.

[2] Qing Y, Guo Y, Zhao Q, et al. Targeting lysosomal HSP70 induces acid sphingomyelinase-mediated disturbance of lipid metabolism and leads to cell death in T cell malignancies[J]. Clin Transl Med, 2023, 13(3): e1229.
